# Stimuli-responsive smart materials enabled high-performance biosensors for liquid biopsies

**DOI:** 10.1186/s12951-025-03541-5

**Published:** 2025-07-01

**Authors:** Xiaoqi Gao, Bayinqiaoge Bayinqiaoge, Ming Li, Rona Chandrawati, Xiangpeng Li, Lining Sun, Chun H. Wang, Chengchen Zhang, Shi-Yang Tang

**Affiliations:** 1 School of Mechanical and Manufacturing Engineering, University of New South Wales, Sydney, NSW 2052 Australia; 2 School of Electronics and Computer Science, University of Southampton, Southampton, SO17 1BJ UK; 3 School of Chemical Engineering, University of New South Wales, Sydney, NSW 2052 Australia; 4 College of Mechanical and Electrical Engineering, Soochow University, Suzhou, 215000 China

**Keywords:** Stimuli-responsive materials, Smart materials, Biosensors, Liquid biopsy

## Abstract

Liquid biopsies have emerged as a key tool that enables personalized medicine, enabling precise detection of biochemical parameters to tailor treatments to individual needs. Modern biosensors enable real-time detection, precise diagnosis, and dynamic monitoring by rapidly analyzing biomarkers such as nucleic acids, proteins, and metabolites in bodily fluids like blood, saliva, and urine. Despite their potential, many biosensors are still constrained by mono-functionality, sub-optimal sensitivity, bulky designs, and complex operation requirements. Recent advances in stimuli-responsive smart materials present a promising pathway to overcome these limitations. These materials enhance biomarker signal transduction, release, or amplification, leading to improved sensitivity, simplified workflows, and multi-target detection capabilities. Further exploration of the integration of these smart materials into biosensing is therefore essential. To this end, this review critically examines and compares recent progress in the development and application of physical, chemical, and biochemical stimuli-responsive smart materials in biosensing. Emphasis is placed on their responsiveness mechanisms, operational principles, and their role in advancing biosensor performance for biomarker detection in bodily fluids. Additionally, future perspectives and challenges in developing versatile, accurate, and user-friendly biosensors for point-of-care and clinical applications using these smart materials are discussed.

**Graphical abstract:**

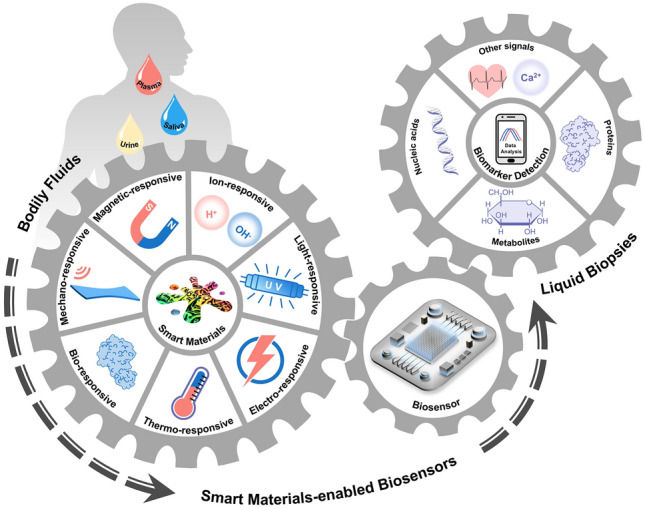

## Introduction

Individual differences in molecular, physiological, and environmental exposure have been demonstrated to induce inter-individual variation in disease processes, making a single cure-all solution unlikely. In this context, personalized medicines tailor treatment strategies for each patient’s unique characteristics by analyzing their physiological functions and biochemical parameters. Emerging approaches, such as DNA sequencing, high-throughput proteomics, advanced imaging techniques, and liquid biopsies have been developed to enable the real-time detection and monitoring of disease-related biomarkers [1–4]. Among these approaches, liquid biopsies, which analyze disease-related biomarkers present in bodily fluids, have recently gained growing attention [5, 6]. Due to the readily accessible sample sources like urine, sweat, saliva, peripheral blood, etc., liquid biopsies provide a minimally invasive, repeatable, and real-time approach to diagnosing diseases and monitoring overall health. Because bodily fluids contain a wide range of biomarkers, such as circulating tumor DNA (ctDNA), exosomes, and specific proteins, liquid biopsies have the potential as a versatile tool for detecting these biomarkers and providing valuable insights into disease status. These advantages establish liquid biopsies as a leading approach for developing emerging diagnostic tools for clinical applications.

The promising clinical applications of liquid biopsies have spurred the development of detection devices for facilitating their routine use in hospitals and at home. The most notable example is a biosensor, which converts the concentration of a biological analyte in a fluidic sample into a measurable signal such as a change in color, fluorescence, or electric current [7]. A biosensor typically consists of three parts: a receptor for recognizing biomarkers, a signal transducer, and a signal analyzer. In a biosensor, the receptor selectively recognizes a target biomolecule, triggering a specific biochemical reaction on the transducer and finally leads to a readable output. Based on the working mechanism of the transducer, biosensors can be categorized into several types, including optical, electrochemical, magnetic, thermal, and piezoelectric [8–12]. These integrated receptor-transducer devices provide specific quantitative or semiquantitative information with high sensitivity, accuracy, and fast turnaround time [13].

Smart materials change their physical or chemical properties in response to external stimuli, including ions, biomolecules, thermal, acoustic, light, electric, and magnetic fields. These stimuli-responses can be harnessed to improve key aspects of detection strategies, such as target release and recognition, as well as signal transduction and reporting. Their integration simplifies operations and enhances biosensing performance compared to conventional biosensors lacking such materials. For example, the receptor functionalized by ion- or bio-responsive materials can specifically recognize multiple target analytes simultaneously without generating interference, enhancing versatility and selectivity of the sensing performance [14–16]. In addition, smart materials that are responsive to light, electrical, or thermal stimuli are capable of simplifying and minimizing the transducing microsystem by triggering various physicochemical or biochemical reactions [13, 17–19]. Their obvious changes in physical properties such as fluorescence and conductivity benefit the signal readout [20, 21]. Mechano- and thermo-responsive materials could also serve as signal amplifiers by controlling the release of biomarkers [22, 23]. Therefore, smart materials bring great benefits, such as enhanced selectivity, versatility, and temporal controllability to biosensors and pave the way for broader applications.

**Fig. 1:**
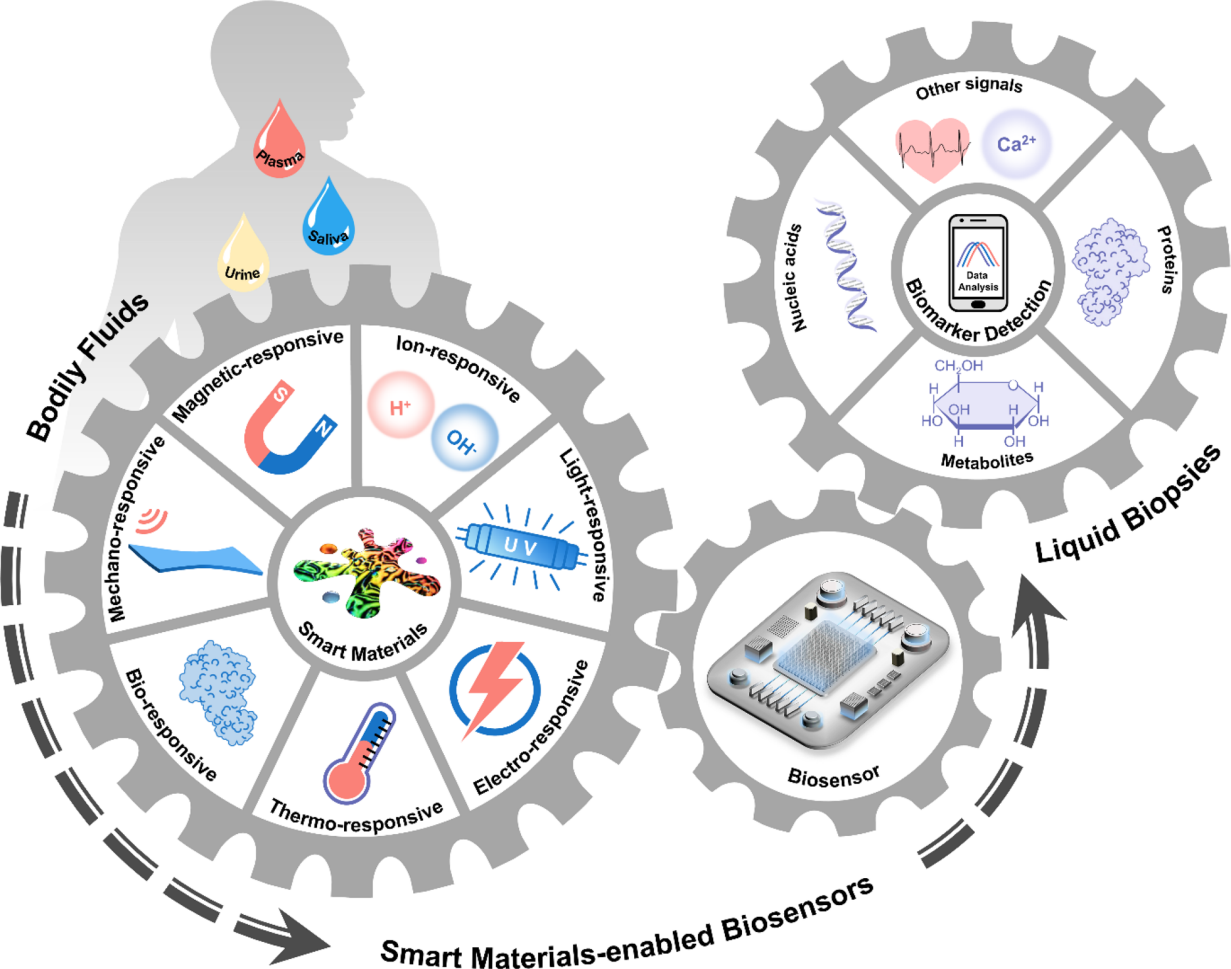
Smart materials enabled biosensors for liquid biopsy and their applications

In this review, we explore how smart materials enhance the performance and expand the application scope of biosensors that are specially designed for detecting biomarkers in bodily liquids (Fig. 1). We provide a systematic overview of biosensors based on emerging smart materials, including mechano-, light-, electro-, magnetic-, thermo-, ion-, and bio-responsive types. Next, we elucidate the responsive mechanisms of these materials and their working principles when integrated into biosensor systems. This review complements other recent reviews on the biomedical applications of smart materials, focusing on the latest developments in the creation of advanced biosensors. We aim to provide researchers with a clear picture and comprehensive understanding of how smart materials can be integrated into biosensing systems and how their stimuli-responsive properties can be harnessed to enhance the performance of biosensors, which can serve as an inspiring reference for further design of versatile and advanced biosensors.

## Mechano-responsive materials enabled biosensors

Mechano-responsive materials, including force-responsive or ultrasound-responsive materials, have gained significant attention for their applications in flexible devices and non-invasive detection in liquid biopsies. In the presence of mechanical forces, piezoelectric materials can generate electrical signals to monitor blood pressure and pulse. As a non-invasive mechanical wave, ultrasound can carry mechanical energy through various mediums. Thus, motion states of smart materials such as nanorobots can be remotely manipulated to increase the plasma level of biomarkers, facilitating downstream analysis. This section summarizes mechano-responsive materials in biosensing, such as micro- and nanobubbles, acoustic nanorobots, piezoelectric materials, and liquid metals.

### Micro- and nanobubbles

**Fig. 2:**
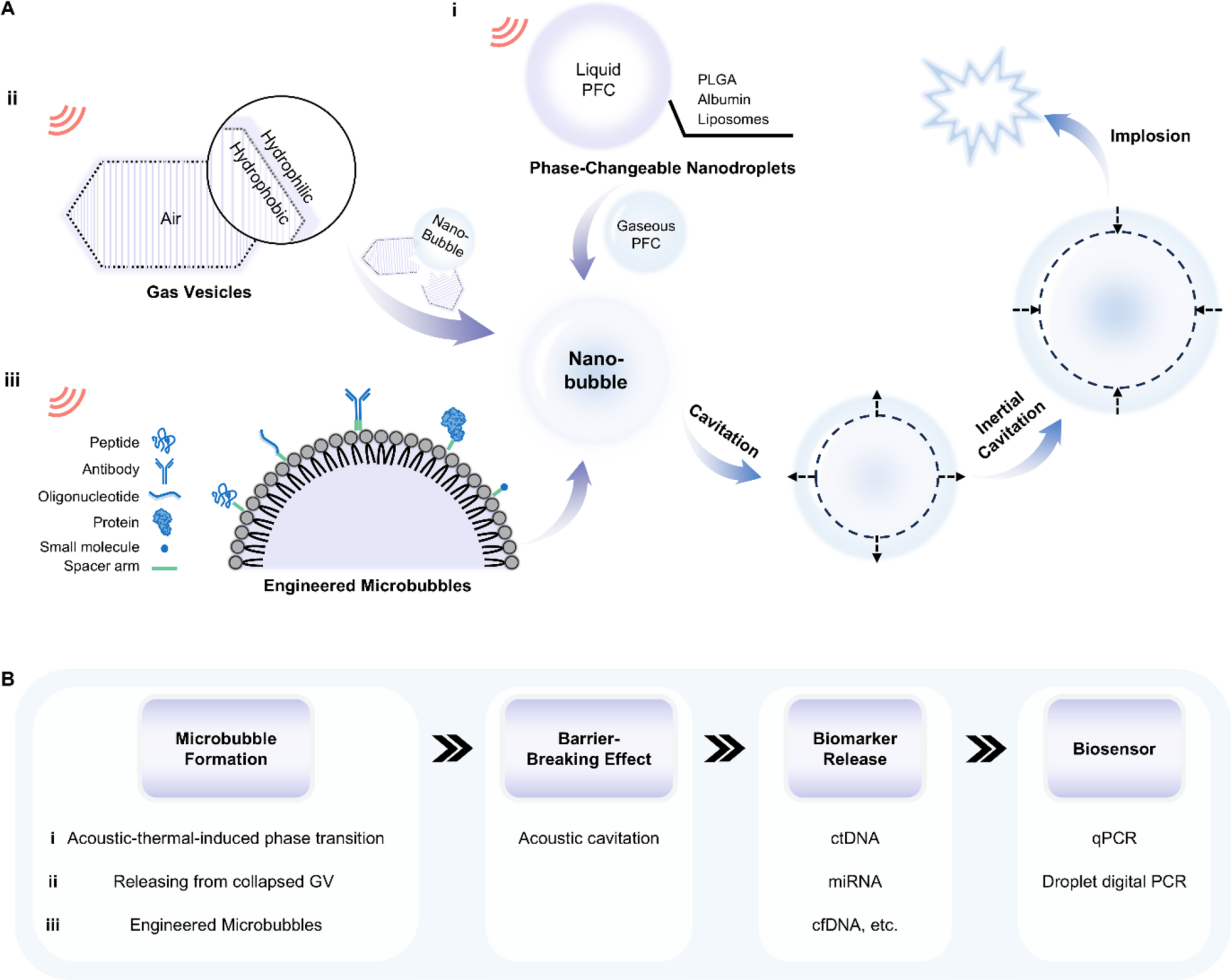
Responsive mechanisms of micro- and nanobubbles under ultrasound stimuli and their working principles when applied in biosensors. **A** Schematic illustration of the generation of micro- and nanobubbles through **i** phase-changeable nanodroplets, **ii** gas vesicles and **iii** engineered microbubbles under an ultrasound field. The generated nanobubbles undergo cavitation and final implosion as acoustic pressure increases. **B** The working principle underlying nanobubbles’ applications in detecting biomarkers in bodily fluids. Their barrier-breaking effect improves detecting sensitivity by facilitating extratumoral biomarker release. PFC: perfluorocarbon; GV: gas vesicles; ctDNA: circulating tumor DNA; miRNA: microRNA; cfDNA: cell-free DNA; qPCR: quantitative polymerase chain reaction

The term micro- and nanobubble usually refers to a hollow nanoparticle filled with a gas wrapped by a layer of functional biomaterials [24]. Depending on their generating mechanism, nanobubbles can be grouped into three categories: phase-changeable nanodroplets, gas vesicles, and engineered microbubbles. Phase-changeable nanodroplets are formed by encapsulating nontoxic perfluorocarbon (PFC) liquid phase with biomaterials such as poly(lactic-co-glycolic) acid (PLGA), albumin and liposomes at the sub-micron scale (Fig. 2A-i) [25]. Due to its high vapor pressure and low surface tension, the PFC liquid phase in the phase-changeable nanodroplets is stabilized at the nanoscale and remains in the liquid state until triggered by ultrasound [26, 27]. When subjected to sufficient acoustic pressure, these nanodroplets undergo a liquid–gas phase transition and transform into nanobubbles. This phenomenon, termed acoustic droplet vaporization (ADV), provides a thermodynamically favorable way to generate nanobubbles in situ, expanding them to diameters up to ten times their original size [28].

Gas vesicles (GVs) are air-filled protein organelles first discovered in cyanobacteria in 1965 [29]. GVs typically adopt cylindrical or spindle-shaped nanostructures with lengths varying from 100 nm to 2 μm and widths ranging from 45 to 200 nm (Fig. 2A-ii). The amphiphilic characteristic of the 3-nm-thick protein shell enables gas to freely permeate in and out of GVs’ hollow nanostructures while keeping inside free of the aqueous phase. When the applied acoustic pressure is above the designed critical collapse pressure of GVs, protein shells are cracked and air inside is released to the surrounding medium, resulting in the formation of nanobubbles. Thus, GVs are expected to serve as seeds for nanobubble production [30]. Besides the indirect generating mechanism above, micro- and nanobubbles can also be manufactured directly. Tiny gas bubbles with a diameter of 1–10 μm are enclosed in the lipid shell, which is covered with ligands that interact with the exterior environment (e.g., proteins, small molecules and cells) (Fig. 2A-iii). [31, 32] These engineered microbubbles are biocompatible and small enough to target a specific region inside capillaries.

The mechanisms and phenomena of nanobubbles responsive to ultrasounds are various, which gives them a position in biomedical applications such as cargo delivery and ultrasound imaging [33, 34]. For the description here and the discussion in the following sections, we will focus exclusively on nanobubbles’ applications in biosensing, their barrier-breaking effect, which indirectly enhances sensing performance by promoting extratumoral biomarker release into bodily fluids [22, 35–38]. As shown in Fig. 2A, obtained microbubbles would periodically oscillate with a relatively small deformation in a process known as stable cavitation if the pressure amplitude of the acoustic field is below the critical cavitation pressure. After that, at sufficiently high amplitudes, microbubbles would undergo rapid growth and violent collapse (also called inertial cavitation) while causing powerful mechanical influences. Although there is no consensus on the cause of the barrier-breaking effect, emerging evidence has demonstrated that this effect is mediated by stable cavitation [33]. By combining this barrier-breaking effect of microbubbles with focused ultrasounds (FUS), sonobiopsy technology has been proposed to help enrich circulating disease-specific biomarkers for noninvasive molecular diagnosis. When FUS active microbubbles are at a targeting site, localized cavitation exerts pressure on cell connections in biological barrier membranes and loosens intercellular tight junctions, enabling a transient increase in permeability to molecules and matter [27]. Therefore, microbubbles can be generated and activated to cavitate at the targeted position in vivo by FUS in a non-invasive manner. Such localized cavitation mechanically breaks biological barriers to release biomarkers such as circulating tumor DNA (ctDNA), microRNA (miRNA) and cell-free DNA (cfDNA) into bodily fluids like blood, improving the accuracy and sensitivity of the subsequent liquid biopsy using real-time quantitative polymerase chain reaction (qPCR) and droplet digital polymerase chain reaction (ddPCR) (Fig. 2B) [39].

**Fig. 3:**
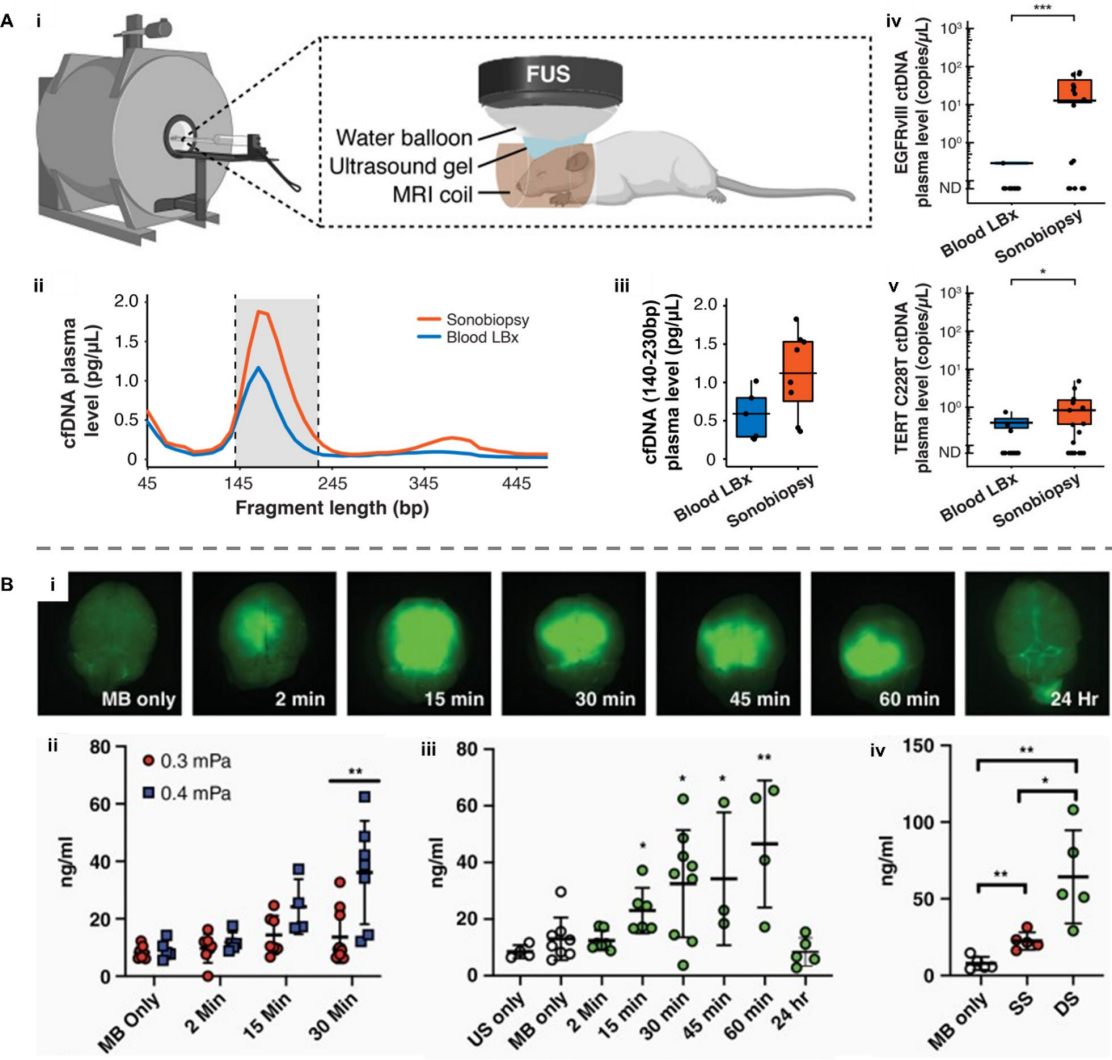
Applications of microbubble-assisted focused ultrasound (FUS)-induced BBB opening in biosensing. **A** Sonobiopsy for minimally invasive detection of glioblastoma-derived ctDNA. **i** The system set up. **ii**–**v** Concentration of ctDNA and cfDNA in plasma after ultrasound treatment. Reproduced with permission [43]. Copyright 2022, Ivyspring International Publisher. **B** Ultrasound-mediated BBB opening for increasing cfDNA plasma level. **i** Representative fluorescent images of cfDNA plasma level at various time points post sonication on mouse head. **ii**–**iv** Optimal acoustic power and optimal blood collection time post sonication. Reproduced with permission [44]. Copyright 2021, Oxford University Press. BBB: blood–brain barrier; cfDNA: cell-free DNA; ctDNA: circulating tumor DNA; US: ultrasound only; MB: microbubble only; SS: single sonication; DS: double sonication

Among all biological barriers, particular attractions are drawn to the blood–brain barrier (BBB), a unique vascular structure characterized by specialized tight junctions. While this endothelial tissue efficiently protects the brain from unwanted metabolites and pathogens, it also prevents brain tumor-derived molecular biomarkers from entering the bloodstream [40]. Such a side-effect leads to poor sensitivity and accuracy of blood-based liquid biopsy (blood LBx) for brain-related diseases due to deficient concentrations of related circulating biomarkers in the blood [33, 41, 42]. To tackle this challenge, Chen et al. developed a FUS-based liquid biopsy (sonobiopsy) technique by combining FUS with microbubbles, providing a complementary approach to improve biomarker sampling and indirectly enhance detection performance [43]. A mouse glioblastoma multiforme (GBM) model was used to compare the plasma levels of cfDNA with sonobiopsy or conventional blood LBx (Fig. 3A-i). After sonobiopsy treatment, the cfDNA concentration in the blood increased (Fig. 3A-ii) and the plasma level of mononucleosomal cfDNA (140–230 bp) was enhanced approximately by twofold compared with blood LBx (Fig. 3A-iii). To further validate the potential for the clinical application of sonobiopsy, ctDNA mutation detection was conducted in a porcine GBM model. The sonobiopsy group showed a 270-fold elevation in the EGFRvIII ctDNA level (Fig. 3A-iv) and a ninefold raise in the TERT C228T ctDNA level (Fig. 3A-v). With ddPCR, sonobiopsy enhances the diagnostic sensitivity for EGFRvIII and TERT C228T from 7.14% to 64.71% and from 14.29% to 45.83%, respectively. This work demonstrated, for the first time, that sonobiopsy improved the detecting sensitivity of two tumor-specific mutations in both mouse and porcine GBM models, paving the way for promoting sonobiopsy to clinical applications.

To obtain a better downstream analysis of glioma-derived biomarkers, Sonabend et al. investigated the optimal variables including collecting time for blood following sonication and FUS parameters in an intracranial glioma mouse [44]. Sodium fluorescein was used to visualize BBB disruption at different time points post sonication ranging from 2 min to 24 h as shown in Fig. 3B-i. The increase in acoustic pressure from 0.3 MPa to 0.4 MPa significantly enhanced the cell-free DNA (cfDNA) concentration in plasma from 13.63 ng mL^−1^ to 36.9 ng mL^−1^ 30 min post-sonication (P = 0.0039, Student’s 2-tailed t-test) (Fig. 3B-ii). Under the same ultrasound parameters, cfDNA concentrations significantly increased at 15 min post-sonication, peaked at 60 min (46.54 vs 13.01 ng mL^−1^ for ultrasound only, P = 0.0027, Student’s two-tailed t-test), and eventually returned to baseline levels by 24 h post-sonication (Fig. 3B-iii). This trend was consistent with the change in fluorescent intensity in Fig. 3B-i. Figure 3B-iv shows that 2 sequential sonication (DS) treatments significantly elevated cfDNA levels compared to single (SS) treatments (64.32 vs 22.54 ng mL^−1^, P = 0.0166, Student’s 2-tailed t-test). This study demonstrated that cfDNA released by FUS-mediated BBB opening into the blood circulation is influenced by time and sonication parameters, providing important considerations for future investigations relative to US-mediated BBB opening-induced enrichment of brain tumor biomarkers.

### Acoustic nanorobots

**Fig. 4:**
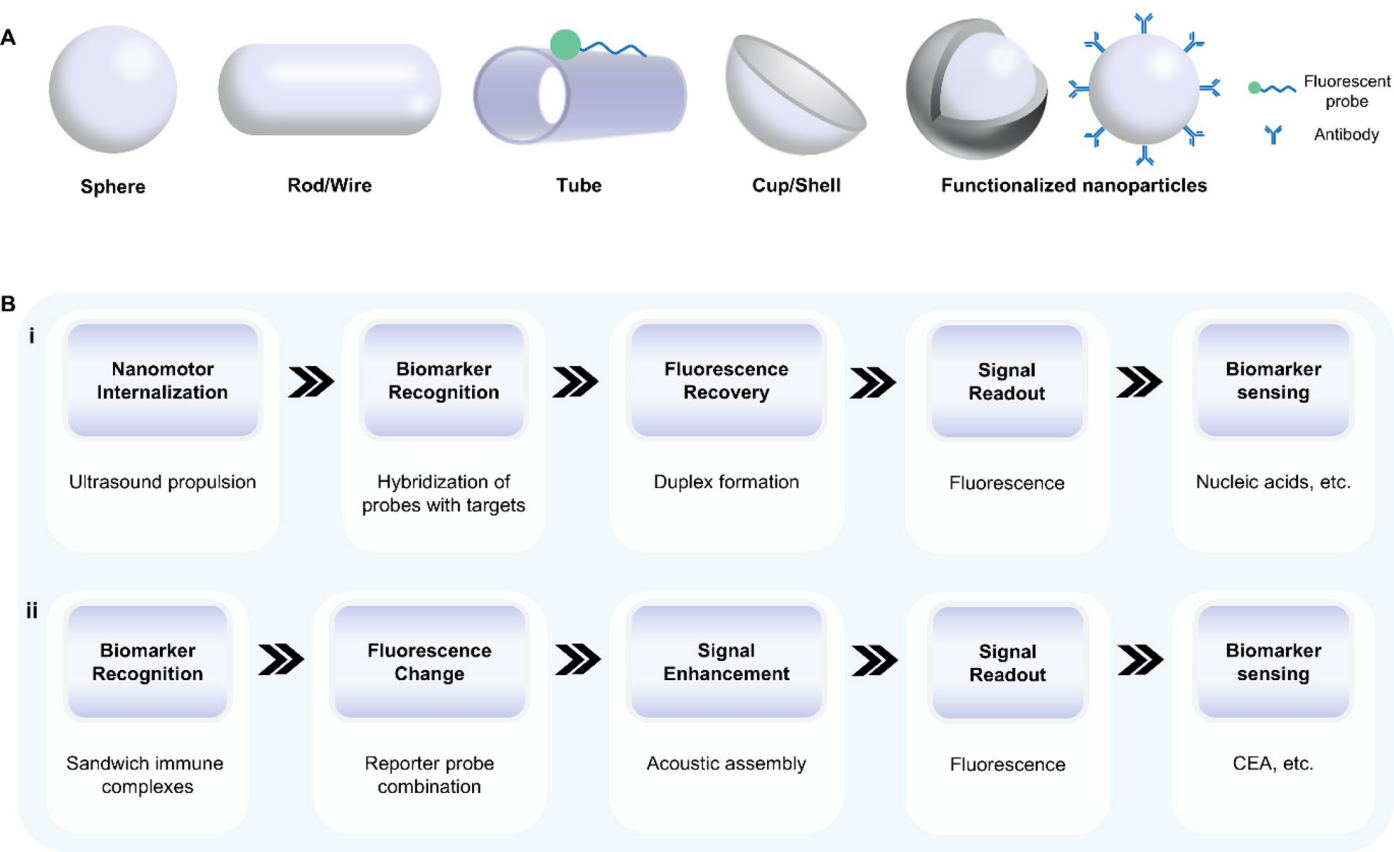
Representative classifications of acoustic robots and their working principles when applied in biosensors. **A** Schematic illustration of the structures of different types of acoustic robots. **B** The working principle underlying the applications of acoustic robots in detecting biomarkers in bodily fluids. **i** Ultrasound propulsion makes functionalized nanoparticles intracellular sensors, and **ii** the acoustic assemble effect enhances detecting sensitivity by amplifying fluorescent signals. CEA: carcinoembryonic antigen

Acoustic nanorobots represent a series of artificial nanomachines that convert acoustic energy into mechanical motions. According to their structure, nanorobots can be roughly divided into four categories: sphere, rod/wire, tube, and cup/shell. Inspired by machines in nature such as vesicular, spermatozoa and bacteria, various functionalized nanoparticles are designed to further improve their stability, biocompatibility, and intake efficiency in vivo (Fig. 4A). In addition to the structure of acoustic nanorobots, careful consideration should also be given to their size and components. The size is related to resonance frequency, motion speed, and bioavailability. Smaller sizes (lower than 200 nm) typically allow nanomotors to have greater propulsion speed and higher efficiency in penetrating deep organs, while larger-sized acoustic nanorobots are more stable and have a larger surface area for immobilizing biomolecules [45]. As for the components, metal materials are currently preferred because they can receive more acoustic radiation than polymers [46]. These design considerations of acoustic nanorobots have been comprehensively discussed in previous review articles [47].

The actuation mechanisms of ultrasound-driven nanomotors vary according to their geometric structures. Nanosphere in an acoustic field is propelled by acoustic radiation force [48]. In this process, the nanosphere is pushed to neighboring pressure nodes (PNs, i.e., points in a standing acoustic wave where the pressure remains minimal or zero) and assembled into a shape consistent with that of acoustic PNs. By modulating the frequency and phase of applied acoustic waves, PNs’ positions in the acoustic wavefield are dynamically changed, and arbitrary motions of the nanosphere in the planer are achieved [49, 50]. Researchers have found that Janus microspheres can twist and partially rotate under ultrasound, which can be explained by the uneven pressures of both sides and the density asymmetry-induced streaming flow on its boundaries [51]. For nanorods with a concave and convex end, the non-uniformity in shape could lead to an uneven distribution of acoustic pressures along the rod, producing the propulsion [52]. Tubular nanorobots are triggered by ultrasound-induced vaporization. Phase change materials (i.e., PFC emulsions) can be loaded inside nanotubes and generate nanodroplets in situ [53]. In the presence of short ultrasound pulses, acoustic microdroplets will vaporize and generate a large amount of energy, which shoots the nanotube in a “bullet-like” manner. This projectile motion based on ultrasound-induced vaporization can achieve a promising average velocity of 6.3 m s^−1^ (about 58,000 body lengths s^−1^) [53]. As for the nanoshell, the motion mechanism is attributed to acoustic streaming induced by both its asymmetric structure and oscillating bubbles. Gas nanobubbles can be trapped and stored inside the cavity of the nanocup, and can be excited to cavitate internally by FUS. This oscillating bubbles-induced streaming allows controlled on-demand propulsion and rotational motion of the nanoshell [54].

**Fig. 5:**
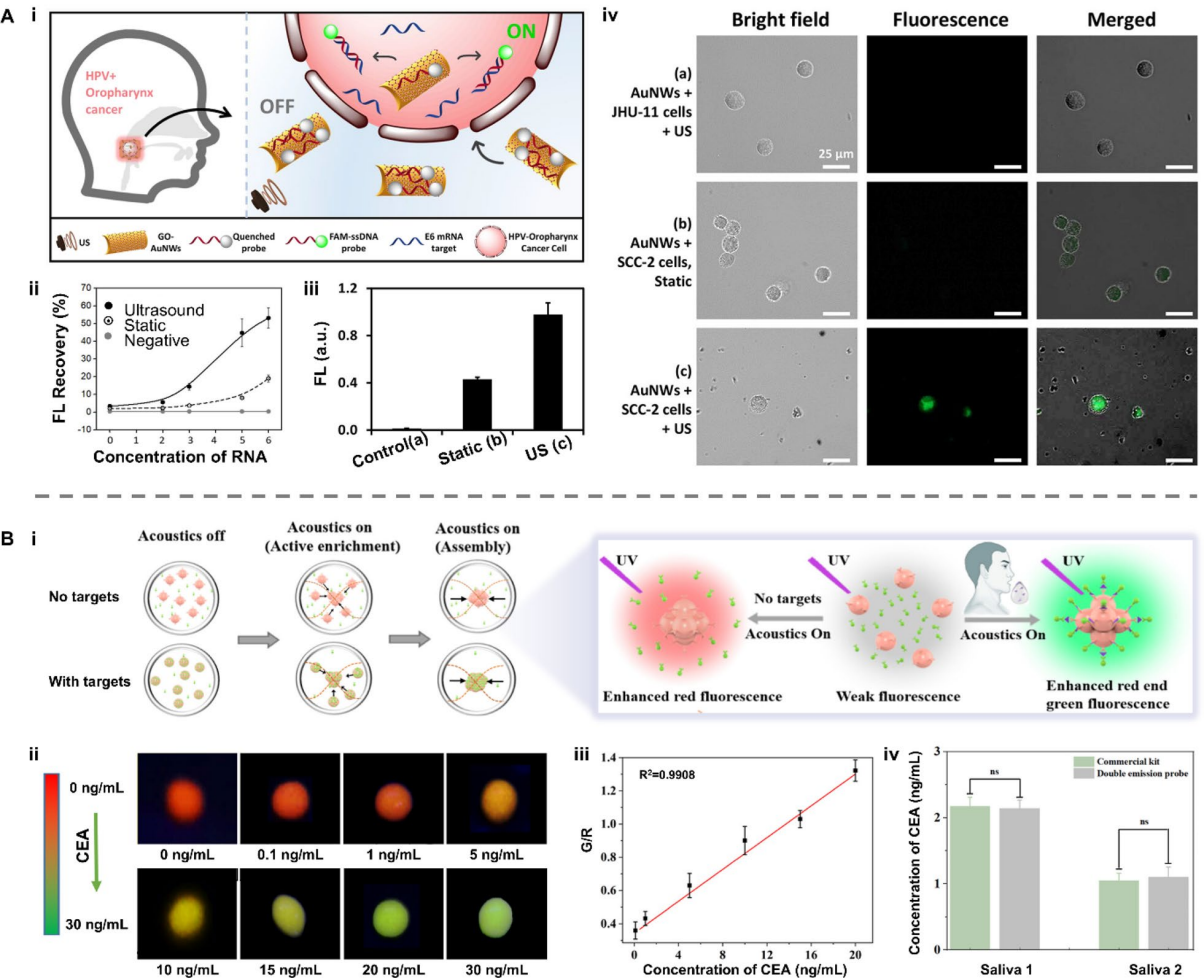
Applications of acoustic robots in biosensing. **A** Acoustic nanomotors for intracellular detection of human papillomavirus-associated head and neck cancer. **i** Working principle. **ii**, **iii** Fluorescent intensity for each condition listed. **iv** Fluorescent images of modified nanomotors after 15-min incubation with **a** HPV-negative or **b**, **c** HPV-positive cells under **b** static conditions or **a**, **c** ultrasound field. Reproduced with permission [61]. Copyright 2019, SAGE Publications Inc. **B** Acoustic aggregation of functionalized nanoparticles to assess the carcinoembryonic antigen (CEA) level in saliva. **i** Working principle of the ratiometric fluorescent platform based on modified Eu-MOFs. **ii** Fluorescent images of modified Eu-MOFs at different concentrations of CEA under acoustic aggregation. **iii** The linear relationship between CEA concentration and the fluorescence ratio of green and red of modified Eu-MOFs. **iv** Comparison of the proposed ratiometric platform and commercial enzyme-linked immunosorbent assay kit for CEA detection of salivary samples. Reproduced with permission [62]. Copyright 2023, American Chemical Society. Eu-MOFs: europium metal–organic frameworks

Based on the above mechanisms, motion modes of nanorobots could be manipulated by ultrasound in a non-invasive and biocompatible way. Therefore, acoustic nanorobots functionalized with fluorescent probes can serve as intracellular sensors to detect biomarkers in real-time (Fig. 4B-i). This “OFF–ON” fluorescent strategy develops the accuracy for sensing biomarkers with an extremely low concentration at the single cell level [55–57]. In addition, through amplifying fluorescent signals by aggregation-induced emission, acoustic-based assembly of functionalized nanorobots is another method to improve detecting sensitivity (Fig. 4B-ii) [58–60]. For example, Califano et al. presented an ultrasound-powered gold nanowire (AuNWs)-based nanomotor for detecting Human papillomavirus (HPV)–associated oropharyngeal cancer (OPC) in vivo [61]. These AuNWs comprised graphene oxide (GO) and dye-labeled single-stranded DNA (ssDNA). This fluorescent probe was quenched by hindering the FRET effect due to the π–π interaction between GO and the dye-labeled ssDNA. In the acoustic field, AuNWs were internalized into human OPC cells and specially combined with HPV16 E6 mRNA, resulting in a fluorescence recovery due to the displacement of the quenched dye ssDNA probe from the surface of AuNWs (Fig. 5A-i). The fluorescence recovery ratio increased with the higher target RNA concentrations, and HPV-positive cells in the ultrasound group showed greater fluorescence recovery at all concentrations compared to static and control groups (Fig. 5A-ii). Incubated with nanomotors, HPV-negative cells as control produced neglectable fluorescence (0.01 au), while HPV-positive cells in the static group produced a detectable signal (0.43 au). After ultrasound treatment for 15 min, HPV-positive cells produced a signal 2.3 times more intense than that in the static group (FI, 0.98 au) due to more efficient nanomotor penetration into cells (Fig. 5A-iii, iv). This work demonstrated the promising application of the nanomotor-based “OFF–ON” fluorescent strategy in HPV-OPC detection in vivo.

As another notable example, Zhang et al. proposed a ratiometric fluorescence platform enhanced by acoustic radiation forces for qualifying carcinoembryonic antigen (CEA) levels in human saliva samples (Fig. 5B-i) [62]. Red-fluorescent europium metal–organic frameworks (Eu-MOFs) conjugated with anti-CEA monoclonal antibody (Eu-MOF-mAb1) nanospheres and green-fluorescent fluorescein isothiocyanate-labeled anti-CEA monoclonal antibody, termed as mAb2-FITC, act as the capture and reporter probes, respectively. In the presence of target CEA, the dual-emission sandwich complex Eu-MOF-mAb1-CEA-mAb2-FITC was formed. As the target CEA increased, the green fluorescence of the dual-emission sandwich complex was dramatically enhanced, leading to a change in the sample fluorescence from the red of the capture probe to the green of the reporter probe. This shift was significantly amplified and enabled visual detection even by the naked eye under ultrasound activation. In the experiments, the fluorescence color of the aggregated nanospheres considerably changed from red to green with the increasing concentrations of CEA (0–30 ng mL^−1^) under a 254 nm UV lamp (Fig. 5B-ii) With the assistance of a smartphone, the ratio value of the green-to-red channel (G/R value) was analyzed, which exhibited a good linear relationship with the CEA concentration in the range of 0.1–20 ng mL^−1^ with R^2^ = 0.9908 (Fig. 5B-iii). In quantifying the CEA concentration in the saliva samples of two volunteers, the results from this integrated ratiometric fluorescence platform were consistent with those from the commercial enzyme-linked immunosorbent assay (ELISA) kit (Fig. 5B-iv). This investigation designed an integrated dual-emission platform and lowered the limit of detection to 0.012 ng mL^−1^ with the help of acoustic-induced aggregation, validating the usefulness of the proposed strategy for clinical and household usage.

### Piezoelectric materials

**Fig. 6:**
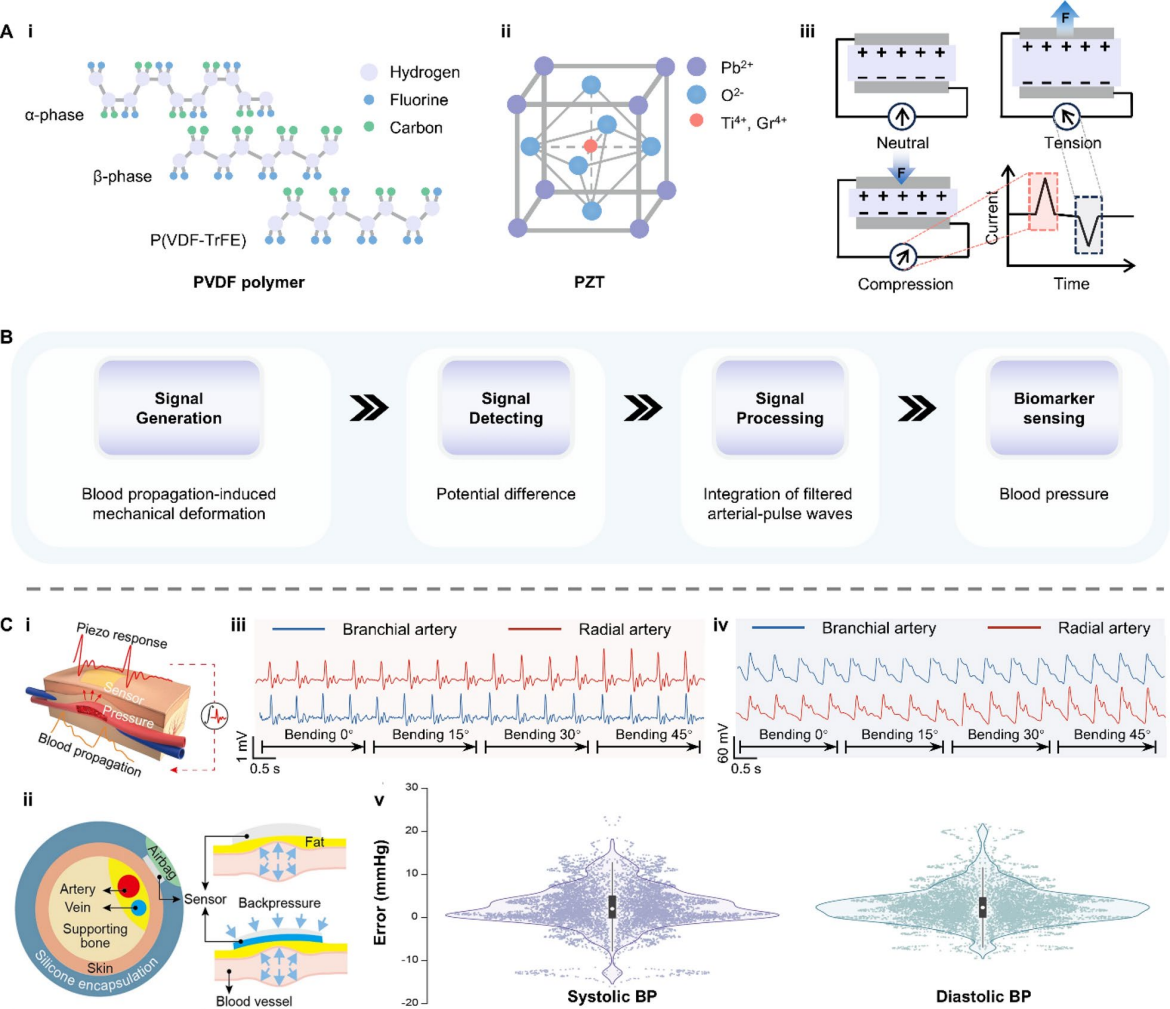
Representative examples of piezoelectric materials and their application in biosensing. **A**-**i**, **ii** Schematic illustrations of two types of piezoelectric materials and **iii** the piezoelectric property. **B** The working principle underlying piezoelectric materials’ application in detecting blood pressure. **C** A thin, soft, miniaturized system (TSMS) using piezoelectric material PZT 5H for continuous wireless monitoring of artery blood pressure. **i**, **ii** Schematic illustration of the blood propagation and generated piezo response. **iii**, **iv** The piezo response and the converted pulse waveform. **v** The BP measurement accuracy of the TSMS compared with commercial CNAP. Reproduced with permission [67]. Copyright 2023, Springer Nature. PVDF: polyvinylidene fluoride; PZT: lead zirconate titanate; BP: blood pressure; CNAP: continuous noninvasive artery pressure

Piezoelectric materials have been receiving increasing attention since it was first proposed by the Curie brothers in 1880 [63]. The piezoelectric effect can be observed in both organic (e.g., polyvinylidene fluoride (PVDF) polymer) and inorganic (e.g., lead zirconate titanate (PZT)) materials with a non-centrosymmetric structure (Fig. 6A-i,ii). Such an asymmetric arrangement of atoms leads to electric dipoles within the material that keep the material electrically neutral when free of mechanical force. However, when piezoelectric materials are subjected to stress, the balance state of electric dipoles is disrupted due to the displacement of atoms or molecules from their original position, and hence net positive and negative charges appear on the opposite sides of the materials (Fig. 6A-iii). Because this conversion from mechanical force to potential change is a molecular phenomenon, piezoelectric materials are sensitive to minor mechanical deformation and can sense blood pressure (BP) and pulse (Fig. 6B) [64–66].

In 2023, Yu et al. reported a thin, soft, miniaturized system (TSMS) for continuous monitoring of arterial BP [67]. This TSMS adopts piezoelectric thin layers (PZT 5H) as the sensors to convert the arterial deformation generated by blood propagation to piezo voltage (Fig. 6C-i). To further increase the mechanical deformation, a micro airbag was built into the wireless wristband to provide powerful backpressure and close-looped feedback for the piezoelectric sensor array (Fig. 6C-ii). Using this TSMS system, the piezo responses of blood propagation in the radial and brachial artery under 0–45° bending deformations were obtained and then processed by a mathematical model to form pulse waveforms (Fig. 6C-iii,iv). After model development, the TSMS system exhibited an accuracy of − 0.05 ± 4.61 mmHg for systolic blood pressure (SBP) and 0.11 ± 3.68 mmHg for diastolic blood pressure (DBP), meeting the Grade A classification according to the British Hypertension Society (BHS) standard. In the measurement accuracy test of this TSMS system, a commercial continuous noninvasive artery pressure (CNAP) monitoring system was chosen as a reference, and continuous blood pressure monitoring for 2 min was conducted on 87 volunteers. The statistical error distribution revealed that most error values were within ± 10 mmHg for both SBP and DBP, demonstrating the TSMS system’s practical utility for precise BP monitoring (Fig. 6C-v). This work validates the feasibility and multifunctionality of fully integrated wearable piezoelectric sensors, paving the way for their popularization of clinical and commercial applications.

### Liquid metals

**Fig. 7:**
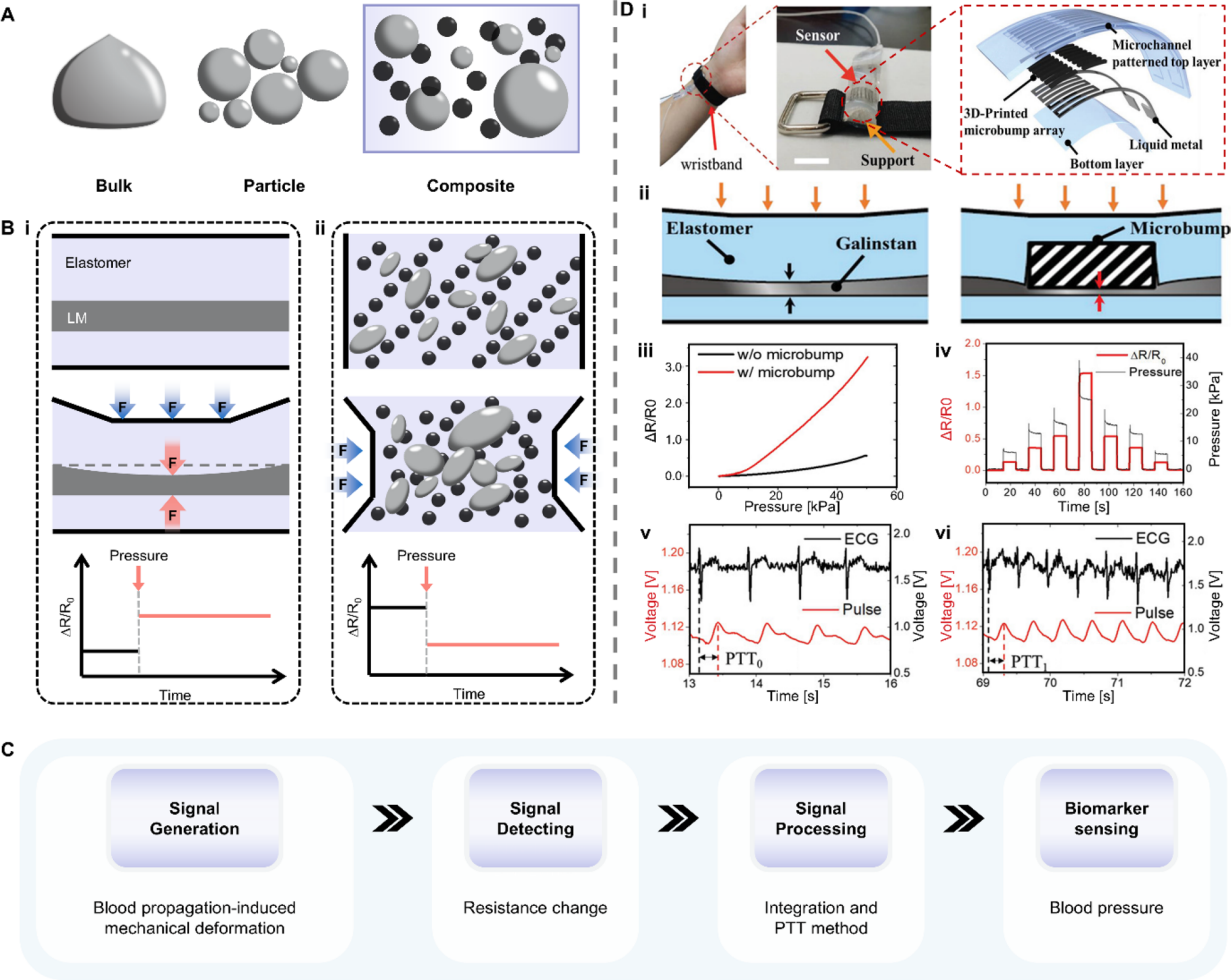
Classifications, responsive mechanisms and typical applications of liquid metal. **A** Liquid metal’s three typical embodiments. **B** Schematic illustrations of the resistance changes of LM composites in response to external stress. **C** The working principle underlying LM’s application in detecting blood pressure.** D** A wearable 3D-printed rigid microbump-integrated LM-based pressure sensor (3D-BLiPS). **i** Schematic view of the proposed 3D-BLiPS. **ii**, **iii** Effect of the microbump on pressure sensitivity. **iv** Dynamic response of the sensor to the application of varying pressure levels. **v**, **vi** Continuous epidermal pulse and ECG signals for PTT calculation before and after exercise, respectively (PTT_0_ = 278 ms, PTT_1_ = 238 ms). The SBP and DBP after exercise were estimated to be 138.4 ± 4.2 and 66.8 ± 1.4 mmHg, respectively. Reproduced with permission [79]. Copyright 2019, Wiley. LM: liquid metal; ECG: electrocardiogram; PTT: pulse transit time; SBP: systolic blood pressure; DBP: diastolic blood pressure

Liquid metals (LMs), a family of emerging smart materials, maintain a liquid state below or near room temperature while offering many unique but useful properties, such as high electrical conductivity, highly controllable surface, and morphological transformability. LMs are currently applied in the biomedical field as three typical embodiments: bulk, particle, and composite [68, 69]. Bulk LM is a single, continuous volume or stream of LM and can be easily broken into LM particles due to its low viscosity. By mixing LM particles with a polymer matrix, flexible LM composites can be obtained (Fig. 7A). Each LM embodiment has its characteristics and distinctive usage and has been reviewed in detail elsewhere [70–73]. Both bulk and flexible LM composites are force-responsive and can express a resistance change when subjected to external stress. The first method for achieving the resistance change is filling LM into a soft elastomer microchannel (Fig. 7B-i). A pressure onto the composite would decrease the cross-sectional area of the microchannel, resulting in a rise in electrical resistance along the microchannel following Ohm's law [74, 75]. The other way is using flexible LM composites, in which LM particles are separated by an elastomer matrix that is inherently an insulator (Fig. 7B-ii). At sufficient pressure, those isolated LM particles will be pushed to connect and form a conductive path, thereby reducing the resistance. This force-responsive property enables LM a promising application in sensing bending-induced pressures such as BP (Fig. 7C) [76–78].

To improve the sensitivity of traditional liquid metal-based pressure sensors, Park et al. proposed a wearable liquid metal-based pressure sensor for cuffless blood pressure estimation (Fig. 7D-i) [79]. A rigid microbump array was integrated into the LM composite to increase the local deformation of the microchannel (Fig. 7D-ii). Results in Fig. 7D-iii and iv) indicate that the LM pressure sensor with a microbump has a better response to pressure compared to the one without a micrbump. Based on the pulse transit time (PTT) method, systolic BP (SBP) and diastolic BP (DBP) were calculated using this LM pressure sensor before and after the exercise, respectively (Fig. 7D-v, vi). After exercise, the estimated SBP and DBP were 138.4 ± 4.2 and 66.8 ± 1.4 mmHg, respectively, compared to 135 and 67 mmHg measured by an automatic digital blood pressure monitor with a cuff. This work demonstrated the significant potential of LM-based pressure sensors for use in electronic skin and other health monitoring applications.

In summary, ultrasonic mechanochemistry leverages the powerful capacity of ultrasound such as its deep penetration in vivo, precise and remote manipulation of mechanophores, and high biocompatibility. Ultrasonically activated micro- and nanobubbles are regarded as promising tools for improving liquid biopsies’ sensitivity by enriching rare analytes in bodily fluids by opening biological barriers. However, this technique requires a high-power and bulky ultrasound system to localize and selectively active micro- and nanobubbles, which limits its general popularization. Acoustic nanorobots can be propelled into cells to achieve single-cell detection, while the pre-treatment time should be further decreased to facilitate practical usage. Although these nanorobots can also be assembled into clusters by acoustic radiation force to enhance fluorescent signal intensity, other acoustics-induced microphenomena (e.g. acoustic streaming) must be carefully curbed for this aim. Wearable electronics based on force-responsive materials can detect blood pressure in real-time. Future research can be expected to expand their biomarker testing range.

## Light-responsive materials enabled biosensors

Light-responsive materials are among the most researched and developed smart materials, which exhibit tunable emission or photoelectric characteristics in response to external light stimuli. These properties are intrinsic in certain metallic and dielectric materials.80 Researchers have also been committed to designing and constructing artificial materials at the molecular level to achieve these light-triggered processes [80, 81]. As light wavelength and intensity can be precisely controlled and the instrument can be easily miniaturized, light-responsive materials play an essential role in the field of analytical chemistry to develop integrated devices for detecting various biochemical substances in bodily fluids. This section introduces four innovative light-responsive materials: artificial enzyme mimics, quantum dots, metal–organic framework, and plasmonic nanoparticles, which commonly serve as signal transducers (i.e., translate the information related to the targeted biomarker into a readable output) or signal amplifiers (amplify and process the output signal) in the optical and photoelectrochemical biosensors.

### Artificial enzyme mimics

**Fig. 8:**
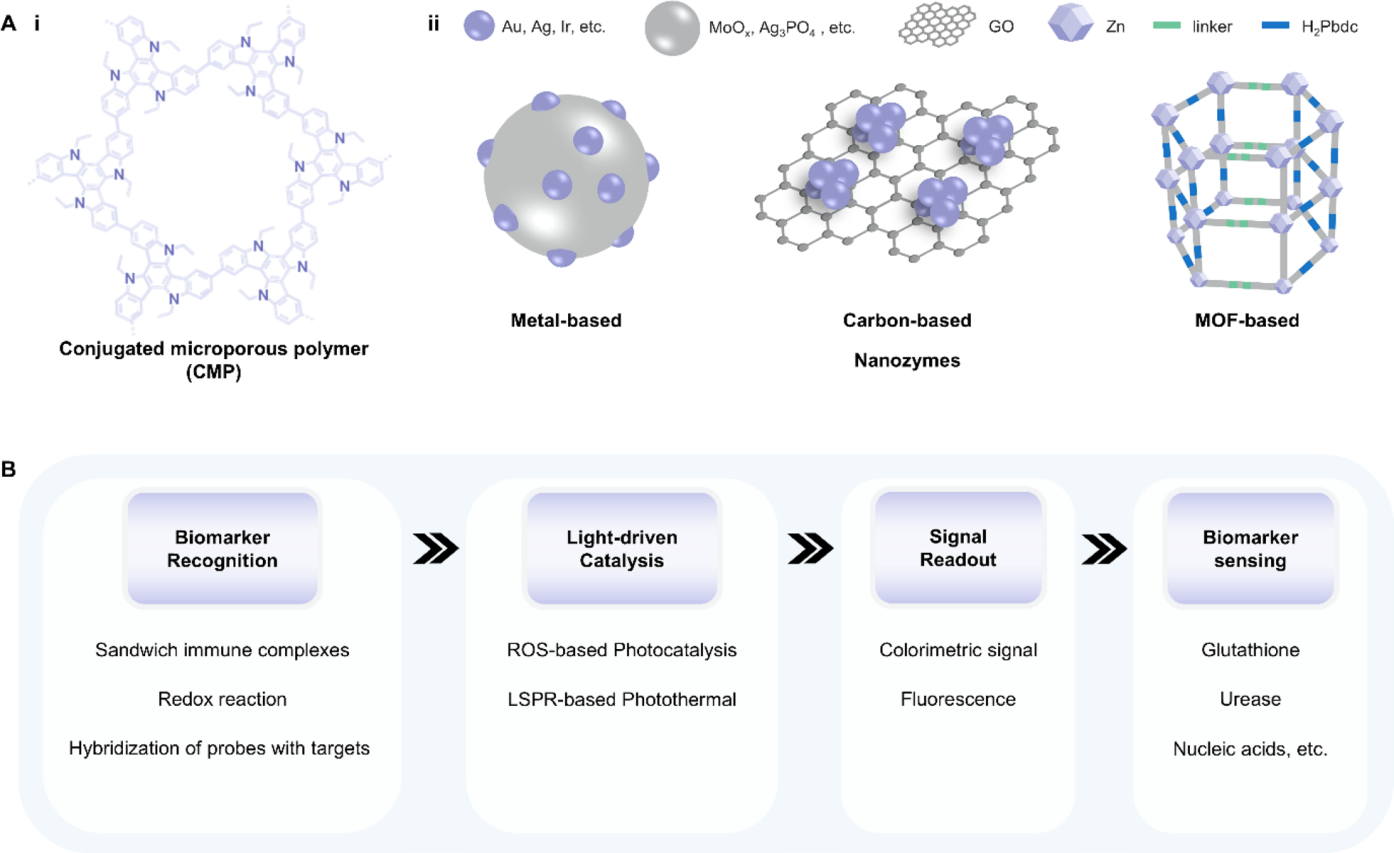
Representative classifications of artificial enzyme mimics and their working principle when applied in biosensors. **A** Schematic illustration of **i** conjugated microporous polymer (CMP) and **ii** nanozymes. **B** The working principle underlying artificial enzyme mimics’ applications in detecting biomarkers in bodily fluids. GO: graphene oxide; MOF: metal–organic framework; ROS: reactive oxygen species; LSPR: localized surface plasmon resonance

Natural enzymes are widespread and participate in various biochemical reactions. As natural catalysts, they could accelerate a process at a rate 10^17^-fold faster than that of an uncatalyzed reaction, offering high catalytic efficiency, selectivity, and stereocontrol [82]. Despite these merits, natural enzymes suffer from poor thermal stability, less versatility toward substrate choice, lack of stability under environmental conditions, and expensive extraction and purification [83]. To address these shortcomings, the artificial enzyme mimics were developed with high stability and reusability. One intriguing branch is the light-responsive artificial enzyme mimics, which incorporate a photo-switchable unit around the active-site mimic to enable reversible catalytic activities under light stimulation. The photo-responsive conjugated microporous polymer (CMP) is a typical example (Fig. 8A-i). This kind of porous material possesses multilevel pore structures, strong light absorption, and high specific surface area, facilitating the generation of reactive oxygen species (ROS) such as superoxide anion (⋅O_2_^−^), hydroxyl radical (⋅OH), and singlet oxygen (^1^O_2_) [84]. Under light irradiation, CMP can efficiently catalyze the oxidation of chromogenic substrates followed by a change in the solution color. This amplifies the output signal and improves the detection accuracy during colorimetric analysis of metabolites in bodily fluids [85–88]. Photocatalytic properties can also be found in some nanomaterials themselves. Nanozymes, particularly those made up of metals, exhibit excellent photothermal conversion efficiency. Benefiting from the large surface area and high electron transfer ability, carbon-based nanozymes can maintain and enhance the catalytic activity of both natural enzymes and nanozymes [89–92]. The intrinsic mesoporous properties of MOF-based nanozymes endow their efficient mass transport for catalysis. (Fig. 8A-ii) [93–95]. Due to the localized surface plasmon resonance (LSPR) effect, nanozymes can generate heat stimulated by light and act as signal transducers in biosensors [96–98].

**Fig. 9:**
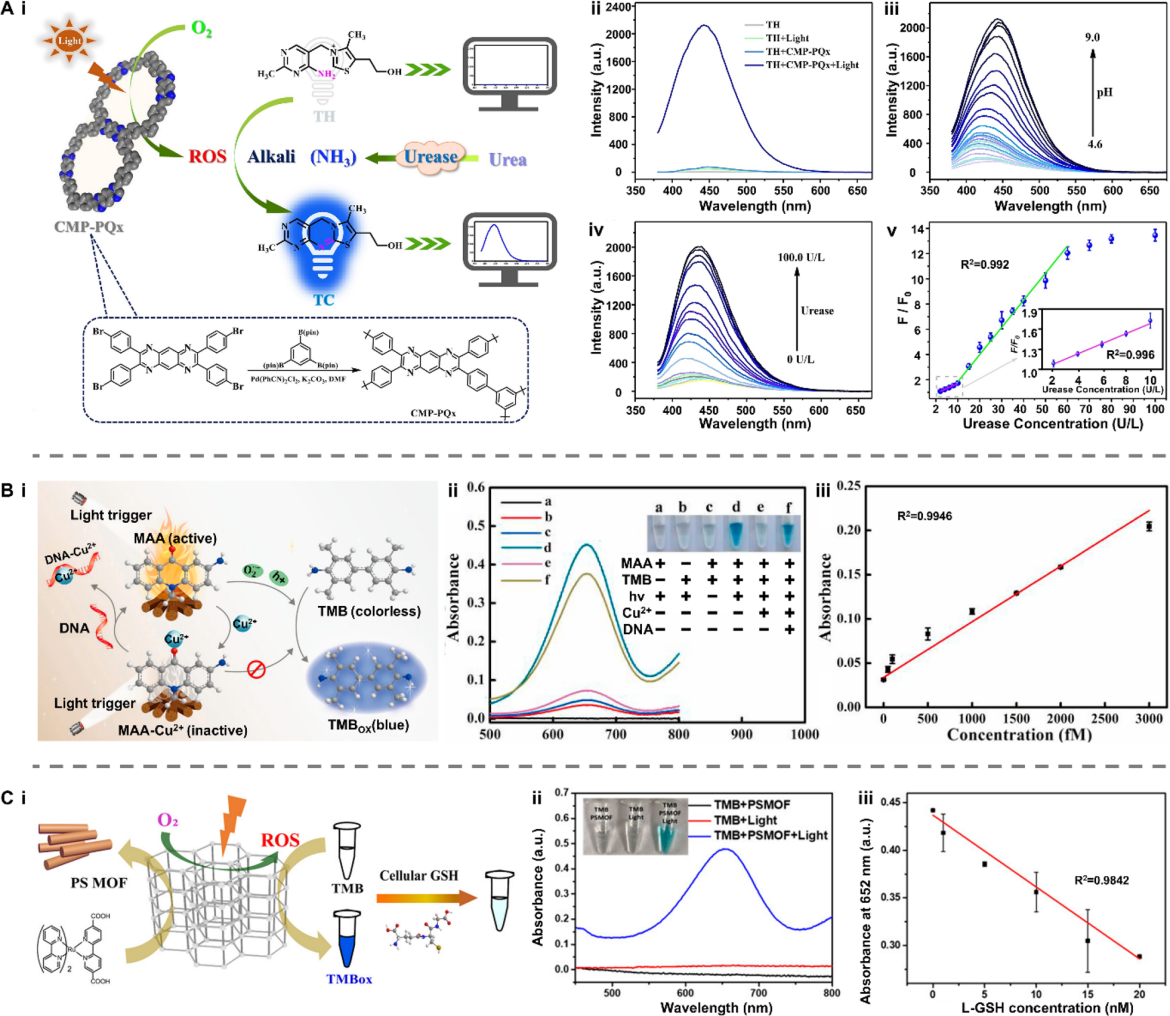
Applications of artificial enzyme mimics in biosensing. **A** Light-responsive oxidase mimic of CMP for urease sensing. **i** The working principle and **ii**–**iv** feasibility of the CMP-PQx-based fluorescent sensor. **v** The linear relationship between urease concentration and the fluorescence intensity ratio (F/F_0_). Reproduced with permission [86]. Copyright 2020, Elsevier. **B** A colorimetric sensor for exosomalmiR-21 detection based on the visible light-triggered oxidase mimic of MAA. **i** The working principle and **ii** feasibility of MAA-based fluorescent sensor. **iii** The linear relationship between exosomal miR-21 concentration and the UV–vis absorbance at 652 nm. Reproduced with permission [102]. Copyright 2021, Elsevier. **C** A photosensitized metal–organic framework (PSMOF)-enabled colorimetric biosensor for cellular GSH detection. **i** The working principle and **ii** feasibility of PSMOF-based colorimetric biosensor. **iii** The linear relationship between GSH concentration and the UV–vis absorbance at 652 nm. Reproduced with permission [93]. Copyright 2019, American Chemical Society. CMP-PQx: CMP containing pyrazino[2,3-g] quinoxaline; MAA: 10-methyl-2-amino-acridone; miR-21: microRNA-21; UV–vis absorbance: UV–visible absorbance; GSH: glutathione

As introduced above, the current photocatalytic mechanism of artificial enzyme mimics can be mainly classified into two types: ROS-based photocatalysis and LSPR-based photothermal effect. Therefore, artificial enzyme mimics are promising tools for amplifying colorimetric signals by oxidizing fluorescence oxidase substrates or for translating changes in targeted concentration into thermal output (Fig. 8B) [99–101]. For example, Su et al. designed a photo-sensitized CMP containing pyrazino[2,3-g]quinoxaline (CMP-PQx)-based fluorescent sensor for quantifying urease in saliva samples (Fig. 9A-i) [86]. The CMP-PQx effectively catalyzed the oxidation of nonfluorescent thiamine (TH) to fluorescent thiochrome (TC) by generating O_2_^·−^ radical in response to visible-light (Fig. 9A-ii). In addition, this oxidation exhibited high pH-responsive performance (Fig. 9A-iii). Thus, a fluorescence sensor was proposed for analyzing urease, which catalyzes the hydrolysis of urea to yield a pH increase and hence a rise in the fluorescence intensity of the CMP-PQx/TH catalytic system (Fig. 9A-iv). The fluorescence intensity ratio (F/F0) has a linear relationship with urease concentration in the ranges of 2.0–10.0 U L^−1^ (R^2^ = 0.996) and 10.0–60.0 U L^−1^ (R^2^ = 0.992), respectively, with a LOD of 0.42 U L^−1^ (Fig. 9A-v). This work introduced the ability of light-responsive CMP as an oxidase mimic for constructing sensors for biological analysis.

More recently, Chen et al. developed a colorimetric sensor to achieve exosomal microRNA-21 (miR-21) detection based on the light-triggered oxidase mimic activity of 10-methyl-2-amino-acridone (MAA) (Fig. 9B-i) [102]. Under the irradiation of visible light, MAA produced photo-induced hole (h^+^) and superoxide anion (O_2_^·−^) to catalyze the oxidation of colorless 3,3’,5,5’-Tetramethylbenzidine (TMB) to blue oxidized TMB (TMB_ox_). Such photocatalytic process was selectively inhibited by Cu^2+^ and then recovered after adding DNA (Fig. 9B-ii). With the help of duplex-strand specific nuclease (DSN)-assisted target recycling amplification, biotinylated DNA capture probes (Cps) hybridized with targeted miR-21 and released the guanine-rich sequence ([G_4_T]_5_) to restore the oxidase mimic activity of MAA hindered by Cu^2+^. This strategy allowed for quantifying the exosomal miR-21 concentration in the range from 50 to 3000 fM, showing a good linear relationship to UV–vis absorbance (R^2^ = 0.9946) with the LOD of 44.76 fM (Fig. 9B-iii). Similarly, Wei et al. presented a photosensitized metal–organic framework (PSMOF) as a colorimetric probe for the detection of glutathione (GSH) in cells (Fig. 9C-i) [93]. This PSMOF catalyzed the oxidation of TMB by formulating ⋅OH and O_2_^·−^ under light simulation, yielding a shift in solution color from colorless to blue (Fig. 9C-ii). This oxidase-like activity of the PSMOF can be inhibited by GSH, resulting in a drop in the characteristic UV–vis absorption of TMB_ox_ as the GSH concentration increases. Inspired by this phenomenon, a colorimetric biosensor was established using the PSMOF/TMB catalytic system, which exhibited a linear relationship between the absorbance at 652 nm and GSH concentration in the range from 0 to 20 μM (R^2^ = 0.9842) with a LOD of 0.68 μM (Fig. 9C-iii).

### Quantum dots

**Fig. 10:**
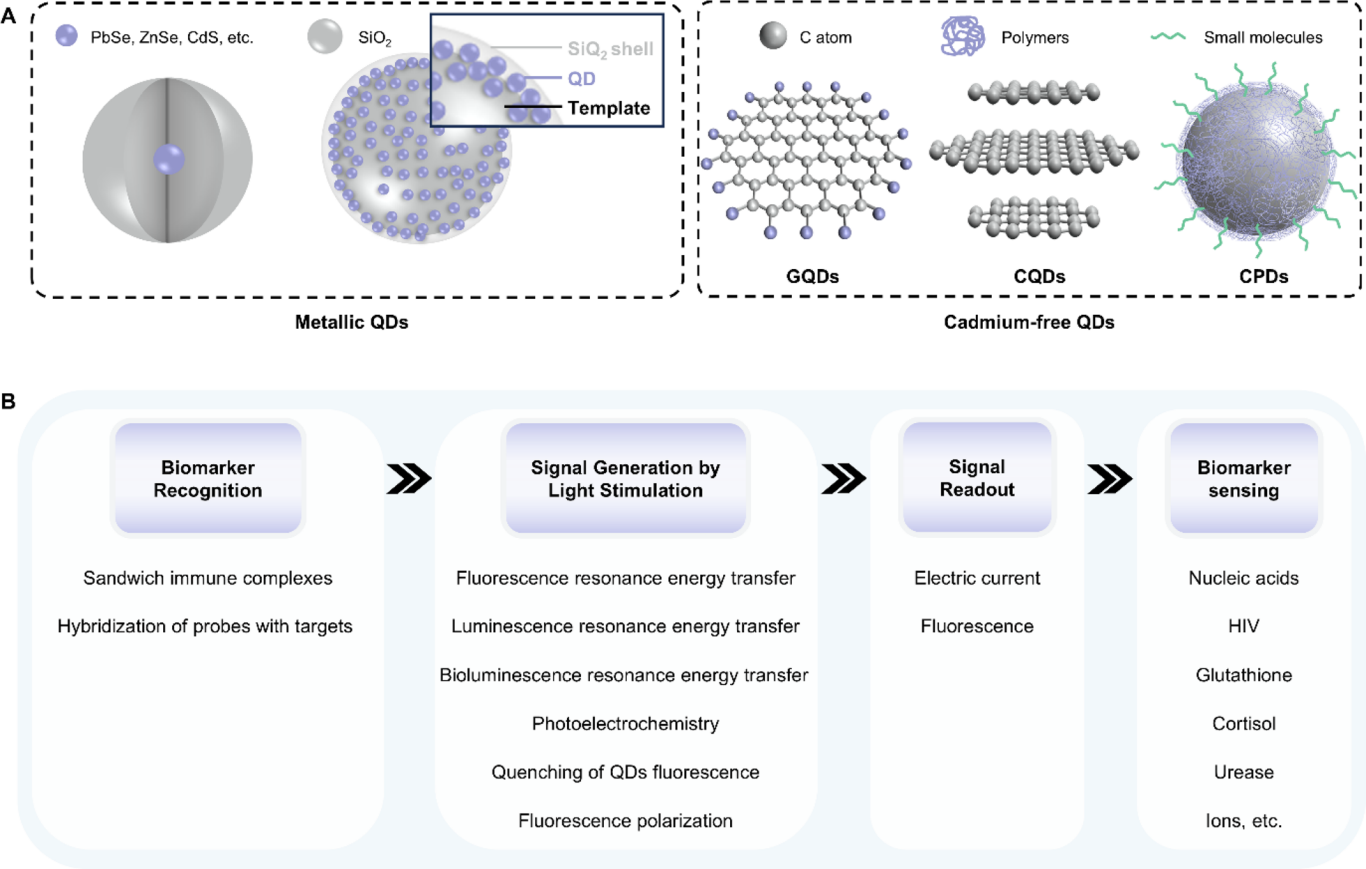
Representative classifications of quantum dots (QDs) and the working principle when applied in biosensors. **A** Schematic illustrations of metallic QDs and cadmium QDs. **B** The working principle underlying QDs’ applications in detecting biomarkers in bodily fluids. QDs: quantum dots; C atom: carbon atom; GQDs: graphene quantum dots; CQDs: carbon quantum dots; CPDs: carbon polymeric dots

Quantum dots (QDs) are a kind of semiconducting nanocrystalline materials with unique optical and electronic properties. According to the component, QDs can be classified into metallic QDs (e.g. SiO_2_-surrounded PbSe, ZnSe, or CdS core materials) and cadmium-free QDs (e.g. graphene quantum dots (GQDs), carbon quantum dots (CQDs) and carbonized polymeric dots (CPDs)) (Fig. 10A) [103–107]. Their optical characteristics are mainly determined by size and structure. For example, QDs with a diameter of 5.0–6.0 nm exhibit orange or red color while smaller QDs with a 2.0–3.0 nm diameter emission blue and green color upon light irradiation [108]. Such size-tunable optical proprieties make QDs attractive materials as fluorescent probes in optical biosensors [109–114]. QD-based fluorescent/bioluminescent biosensors typically involve techniques as follows: fluorescence resonance energy transfer (FRET), luminescence resonance energy transfer (LRET), bioluminescence resonance energy transfer (BRET), fluorescence polarization (FP) and quenching of QD fluorescence (Fig. 10B).

FRET is a non-radiative energy transfer process from a fluorescence donor to an adjacent fluorescence acceptor at a distance of < 10 nm [115]. In the presence of targeted biomarkers, the FRET process is initiated by the formation of the donor–acceptor pair closer enough to achieve dipole–dipole interactions, resulting in a change in fluorescence signal under light irritation [116, 117]. FRET-based biosensors necessarily incorporate an external light source, which is unfavorited for integration and minimization. To address this limitation, LRET is proposed by replacing the fluorescence donor with a luminophore to generate emission light to stimulate the fluorescence acceptor. Similarly, BRET is also a potential alternative to FRET, where bioluminescent luciferase is chosen as the energy donor [118]. In the above-mentioned RET-based biosensors, QDs can act as donor fluorophores or acceptor fluorophores, offering advantages such as high brightness, photostability, and detective sensitivity [119–121]. Fluorescence polarization (FP) is a phenomenon in which the intensity of emission light from a fluorophore varies along different axes of polarization. Such fluorescence anisotropy is inversely proportional to the molecular rotation, which is influenced by the size and weight of the fluorophore [122, 123]. When exposed to light, the interactions between QDs and specific target analytes could be investigated by calculating the emission intensity parallel and perpendicular to the polarization plane of the excitation light. As for biosensors based on the quenching of QD fluorescence, the essence is to hinder the charge transfer between excited QDs and acceptors or disrupt the formation of a close donor–acceptor pair [124, 125].

In addition, QDs can also serve as photoactive materials in photoelectrochemical (PEC) sensors (Fig. 10B). Upon light illumination, electron–hole pairs are generated at the QDs’ surface. Then the generated electrons move to a positively charged electrode/solution-soluble electron acceptor, forming an anodic/cathodic photocurrent [126]. Through this process, the chemical information from a specific biomolecules-induced biorecognition reaction is successfully converted into a photoelectrical current. Similar to size-tunable optical proprieties, the bandgap of the QDs can be adjusted by their size. As the size of QDs reduces, the energy difference between energy bands rises, resulting in discrete energy and a larger band gap. This characteristic can be used for multichannel detection to enhance the detection efficiency of QDs-based PEC sensors. Combining other advantages such as narrow emission spectra, high photoconversion efficiency, and easy surface modification, QDs are considered promising alternatives to organic fluorophores in PEC sensors [127, 128].

**Fig. 11:**
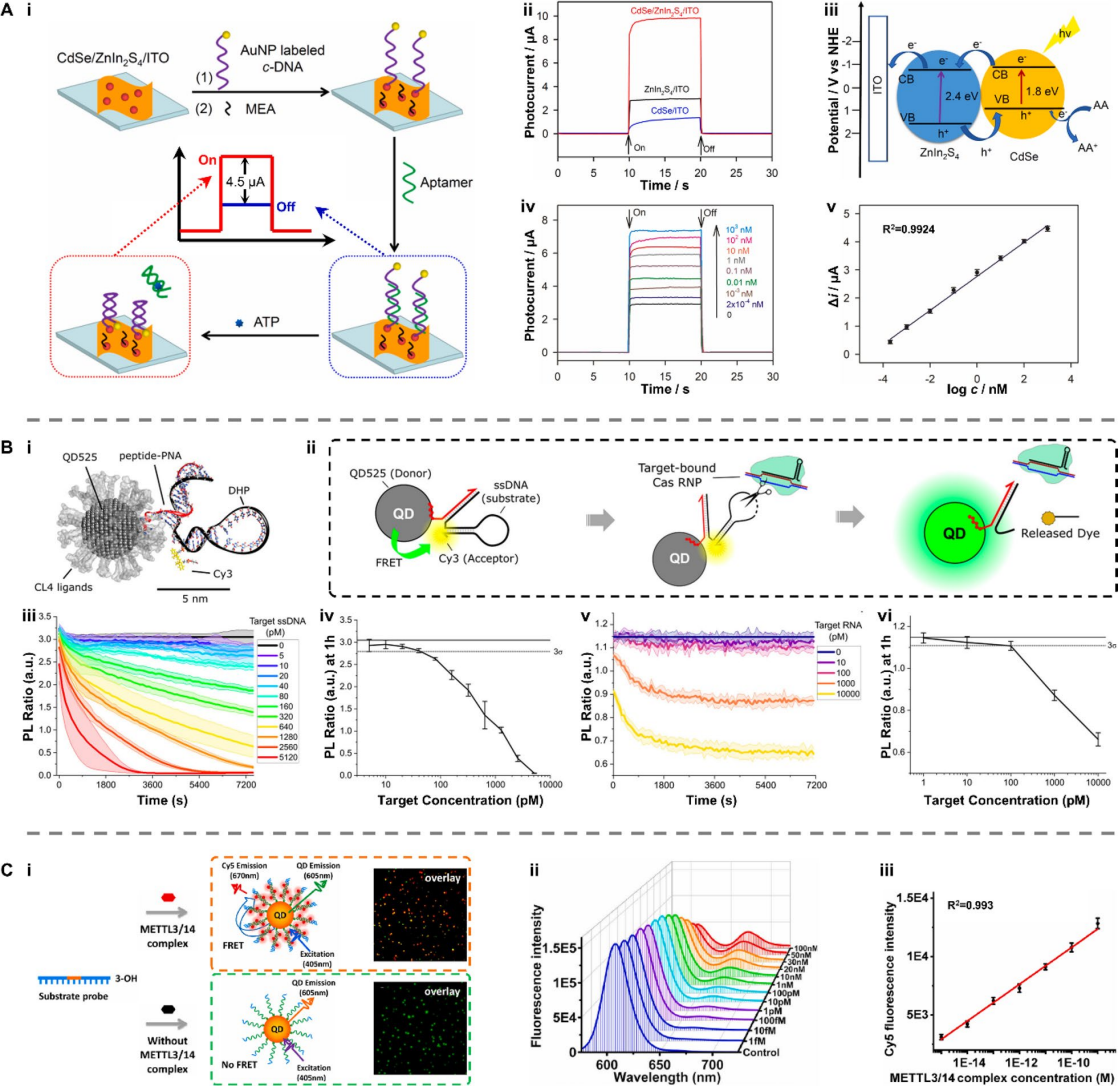
Applications of QDs in biosensing. **A** A photoelectrochemical biosensor using CdSe QDs-decorated ZnIn_2_S_4_ nanosheets for ATP detection. **i** The working principle and **ii**–**iv** feasibility of the CdSe/ZnIn_2_S_4_-based PEC sensor. **v** The linear relationship between the logarithm of the ATP concentration and the photocurrent change. Reproduced with permission [129]. Copyright 2021, Elsevier. **B** QD-based molecular beacons for quantitative detection of nucleic acids. **i** Schematic illustration of the designed fluorescent probe, QD525/DHP-Cy3 complex and **ii** the working principle of the fluorescent biosensor. The concentration of **iii**, **iv** ssDNA and **v**, **vi** lcrVRNA was determined by detecting the PL ratio of Cy3 to QD525. Reproduced with permission [130]. Copyright 2022, American Chemical Society. **C** A single QD-based biosensor for detection of METTL3/14 complex activity in breast cancer tissues. **i** The working principle and **ii** feasibility. **iii** The linear relationship between the logarithm of the METTL3/14 complex concentration and the Cy5 fluorescence intensity. Reproduced with permission [131]. Copyright 2023, Elsevier. PEC: photoelectrochemical; CdSe QDs: lead selenide quantum dots; ZnIn_2_S_4_: zinc indium sulfide; ATP: adenosine triphosphate; DHP: DNA hairpin; QD525: CdSe/CdS/ZnS core/shell/shell quantum dot with an emission peak at 528 nm; PL: photoluminescence

For example, Xie et al. reported a “signal-on” PEC biosensor based on lead selenide (CdSe) QDs-decorated zinc indium sulfide (ZnIn_2_S_4_) nanosheets for detecting adenosine triphosphate (ATP) (Fig. 11A-i) [129]. Under visible-light irradiation, the CdSe QDs-modified ZnIn_2_S_4_ showed a higher PEC activity compared with CdSe QDs and ZnIn_2_S_4_ nanosheets (Fig. 11A-ii,iii). To construct the PEC biosensor, CdSe/ZnIn_2_S_4_/ITO was modified with AuNP labeled complementary DNA strand (c-DNA), which formed double-stranded DNA after hybridizing with aptamers. This double-stranded DNA acted as a spacer to reduce the photocurrent by increasing the distance between CdSe QDs and AuNPs, thereby inhibiting exciton energy transfer between them. In the presence of ATP, the aptamer was dissociated from the double-stranded DNA. As a result, the PEC phenomenon can be recovered due to the close contact between AuNPs and CdSe QDs. The photocurrent of the aptamer/monoethanolamine (MEA)/AuNP-c-DNA/CdSe/ZnIn_2_S_4_/ITO system raised accordingly at rising ATP concentrations (Fig. 11A-iv). A good linearity was found between the logarithm of the ATP concentration ranging from 2 × 10^−4^ to 100 nM and the photocurrent change (R^2^ = 0.9924) with a LOD of 0.1 pM (Fig. 11A-v).

Díaz et al. designed a QD-FRET reporting complex to quantitatively analyze nucleic acids [130]. This strategy utilizes a chimeric peptide-peptide nucleic acid (peptide-PNA) to conjugate dye-labeled nucleic acid hairpins to ZnS-coated QDs, where QD525 and Cy3 acting as FRET donor and acceptor, respectively (Fig. 11B-i). Here, QD525 referred to a CdSe/CdS/ZnS core/shell/shell QD with an emission peak near 528 nm. QD525/DNA hairpin (DHP)-Cy3 complex emitted the fluorescence of Cy3 under 350 nm excitation because of the FRET. While exposed to targeted nucleic acids, the Cy3 dyes were released from the complex, disrupting the closer contact between the donor and the acceptor. Therefore, FRET was curbed and only fluorescence of QD505 was detected (Fig. 11B-ii)). Based on the method, the concentration of ssDNA and lcrV RNA was determined by detecting the photoluminescence (PL) ratio of Cy3 to QD525 (Fig. 11B-iii-vi). These ratiometric reporters were capable of pM target detection with a LOD of 50 pM and 100 pM for target DNA and RNA, respectively. More recently, Zhang et al. proposed a single QDs-FRET biosensor to measure the METTL3/14 complex activity in a single cell [131]. The METTL3/14 complex served as the trigger to initiate FRET between the QD605 donor and Cy5 acceptor by facilitating the formation of the QD605-double-stranded DNA (dsDNA)-Cy5 nanostructure, resulting in an increase in the fluorescence intensity of Cy5 when illuminated by a 405 nm laser (Fig. 11C-i). The Cy5 fluorescence intensity improved as the METTL3/14 complex concentration increased (Fig. 11C-ii), showing an excellent linear dependence on the logarithm of the METTL3/14 complex concentration from 1.0 × 10^−15^ to 1.0 × 10^−9^ M (R^2^ = 0.993) with a LOD of 3.11 × 10^−17^ M (Fig. 11C-iii).

### Metal–organic framework

**Fig. 12:**
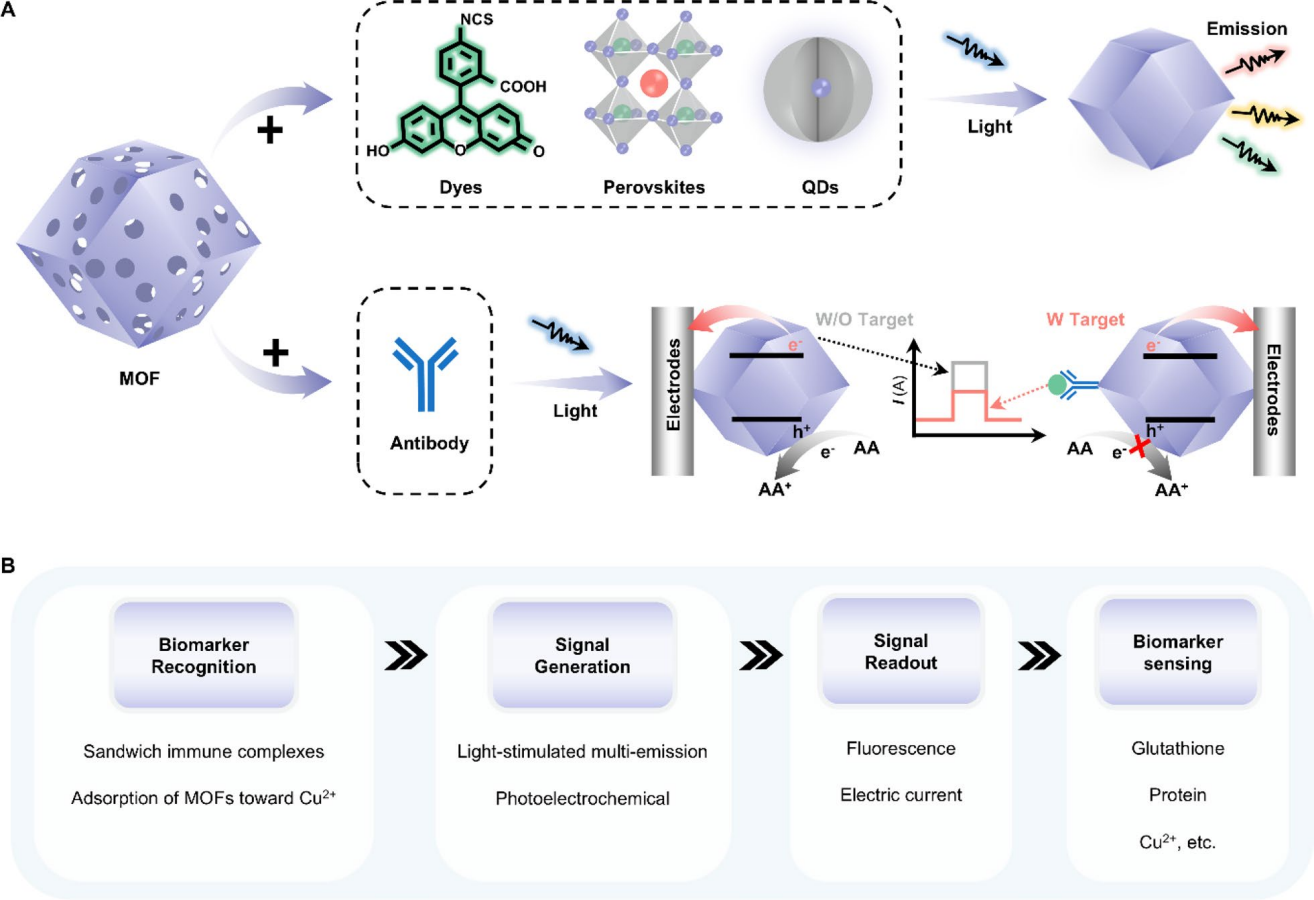
Representative classifications of light-responsive MOF and their working principles when applied in biosensors. **A** Schematic illustrations of luminescent MOFs for fluorescent biosensors and non-luminescent MOFs for PEC biosensors. **B** The working principle underlying MOFs’ applications in detecting biomarkers in bodily fluids. MOF: metal–organic framework

Among all porous materials, metal–organic frameworks (MOFs) have received considerable attention due to their extraordinary porosity and surface area. This porous structure allows the construction of light-responsive MOFs by encapsulating functional guests into MOF cavities. Luminescent MOF (LMOF) is a typical example, which emits fluorescence under light stimulation (Fig. 12A-i). The diversity of luminescent particle (LP) guests, such as fluorescent dyes, perovskites, and QDs, effectively broadens the functionality and application in luminescence sensing of host–guest LMOFs [132]. MOFs can also be used in PEC sensors as photoactive materials or signal-amplifying molecules due to the excellent mass transfer properties enabled by their ultra-high porosity (Fig. 12A-ii) [133].

The principle of LMOF-based biosensors is similar to that of QDs-based fluorescent biosensors, as described in Section “Quantum dots”. In brief, the concentration of biomarkers in bodily fluids is determined by the change in the fluorescence intensity of LMOFs due to the targeted analyte-induced fluorescence enhancement/quenching (Fig. 12B) [134]. MOF-based PEC biosensors focus on effectively converting specific biomarker concentrations into current signal through the redox reaction between electrochemical active species in solution and photoexcited materials upon light irradiation [132]. Thus, the core is to design suitable MOFs to modulate the charge and energy transfer for the PEC reaction. For example, when exposed to specific biomolecules, molecular binding interaction occurring on MOFs would induce steric hindrance, which decreases photocurrent signals by suppressing the diffusion of electron donor/acceptor to the MOFs [133]. Other strategies, including competitive electron transfer, regulation of distance between the signal label and modified electrode, and consumption of electron donor/acceptor, were comprehensively summarized in recent reviews [17, 133, 135].

**Fig. 13:**
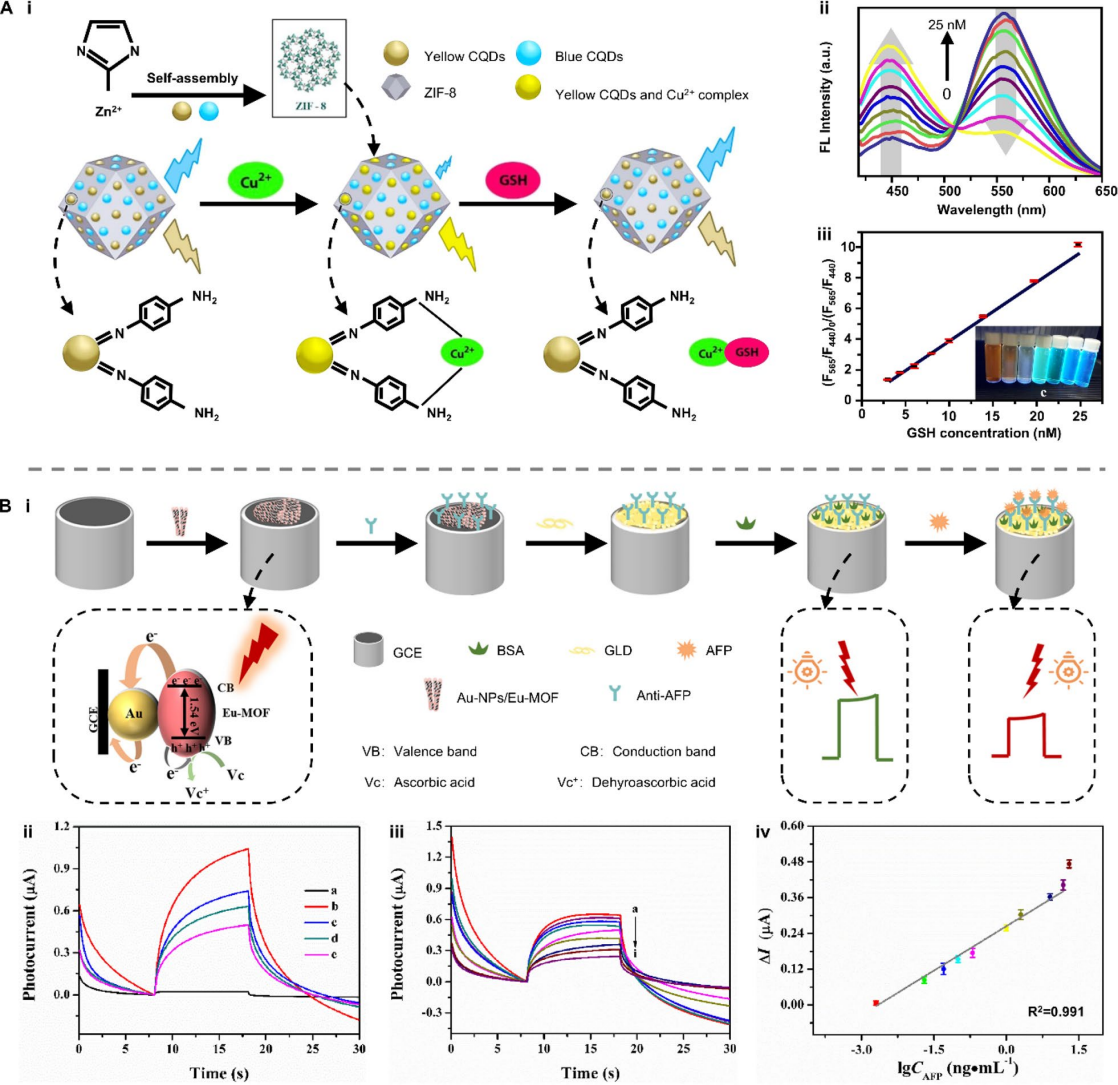
Applications of light-responsive MOFs in biosensing. **A** A dual-emissive MOF-biosensor for ratiometric detection of GSH. **i** The working principle and **ii** feasibility of the MOF-based fluorescent biosensor. **iii** The linear relationship between the logarithm of the GSH concentration and the quenching efficiency, defined as [(F_565_/F_440_)_0_/(F_565_/F_440_)]. Reproduced with permission [140]. Copyright 2019, Elsevier. **B** A Eu-MOFs enabled PEC biosensor for AFP detection. **i** Schematic illustration of the working principle. **ii** The photocurrent responses for the PEC immunosensing interface assembling: **a** a bare GCE, **b** an Eu-MOF@AuNPs/GCE, **c** an anti-AFP/Eu-MOF@AuNPs/GCE, **d** an anti-AFP(BSA)/Eu-MOF@AuNPs/GCE and **e** an AFP/anti-AFP(BSA)/Eu-MOF@AuNPs/GCE. PEC responses of the immunosensor. **iii** The concentration of AFP from a to i: 0.002, 0.02, 0.05, 0.1, 0.2, 1.0, 2.0, 8.0, 15.0 ng mL^−1^. **iv** The linear relationship between the logarithm of the AFP concentration and the photocurrent decrement Δ*I*. Reproduced with permission [141]. Copyright 2022, Elsevier. GSH: glutathione; CQDs: carbon quantum dots; AFP: alpha-fetoprotein; Eu-MOFs: Europium-based metal organic frameworks; GCE: glassy carbon electrode; BSA: bovine serum albumin; AuNPs: gold nanoparticles

Consequently, light-responsive MOFs are ideal materials for the construction of biosensors [136–139]. For instance, Luque et al*.* designed a dual-emissive MOF-biosensor to quantitively analyze glutathione (GSH) (Fig. 13A-i) [140]. Two types of QDs were encapsulated into the zeolitic imidazolate framework (BYCDs@ZIF-8) to fabricate the ratiometric probe, which emitted blue and yellow fluorescence upon excitation at 365 nm. The intensity of blue fluorescence can be quenched by Cu^2+^ without affecting the intensity of yellow fluorescence. In the presence of GSH, the blue fluorescence of the Cu^2+^- BYCDs@ZIF-8 system was recovered (Fig. 13A-ii). The quenching efficiency, defined as [(F_565_/F_440_)_0_/(F_565_/F_440_)], exhibited a good linear relationship with GSH concentration in the range of 3–25 nM with a LOD of 0.9 nM (Fig. 13A-iii). This dual-emissive MOF ratiometric probe enabled the detection of GSH at subnanomolar levels. To enhance the PEC performance, Wang et al*.* combined the intrinsic merits of europium-based metal organic framework (Eu-MOFs) with the outstanding conductivity and local surface plasmon resonance (LSPR) of gold nanoparticles (AuNPs) for sensing alpha-fetoprotein (AFP) (Fig. 13B-i) [141]. Under white light irradiation, Eu-MOF@AuNPs with anti-AFP attachment exhibited a specific photocurrent response. This photocurrent signal was curbed due to the steric hindrance induced by the immunocomplexes of anti-AFP and AFP (Fig. 13B-ii). As a result, the photocurrent gradually decreased with increasing AFP concentration (Fig. 13B-iii). The photocurrent decrement (*ΔI*) and the logarithm of AFP concentrations (lg*C*_AFP_) exhibited a linear relationship (R^2^ = 0.991) with a LOD of 0.16 pg mL^−1^.

### Plasmonic nanoparticles

**Fig. 14:**
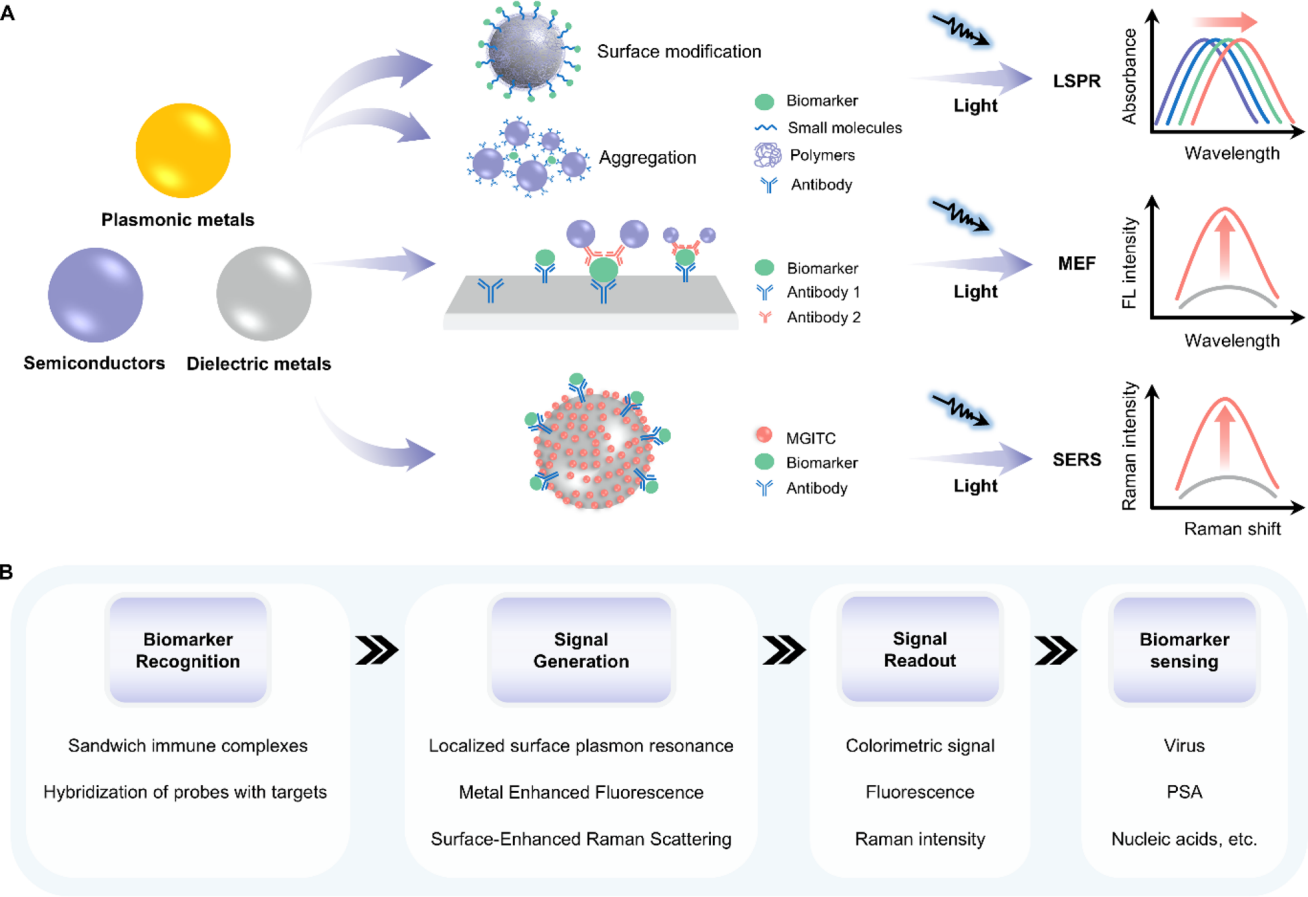
Representative classifications of plasmonic nanoparticles and their working principle when applied in biosensors. **A** Schematic illustrations of three optical phenomena when plasmonic nanoparticles are encountered with biomolecules. **B** The working principle underlying plasmonic nanoparticles’ applications in detecting biomarkers in bodily fluids. LSPR: localized surface plasmon resonance; MEF: metal-enhanced fluorescence; SERS: surface-enhanced Raman scattering; PSA: prostate-specific antigen

Plasmonic nanoparticles (NPs) are another important category of light-responsive materials. These NPs are mainly made of plasmonic metals, semiconductors, and dielectric metals in diverse structures at the nanoscale, displaying optical, electrical, and catalytic properties that are significantly different from those of the bulk counterparts (Fig. 14A) [142]. For example, under an external illuminating light, a collective oscillation of free electrons occurs on the surface of plasmonic NPs due to their large specific surface areas and space restriction on free electrons [143]. When the frequency of the incident light coincides with the inherent frequency of the free electrons, the resonance is formed, which is termed localized surface plasmon resonance (LSPR). LSPR generates plasmonic resonance peaks in the absorption spectra [144]. The peak wavelength depends on the morphology, size and composition of plasmonic NPs [145]. Therefore, based on LSPR, plasmonic nanoparticle-nanoparticle interactions can be characterized by the absorbance shift. LSPR also enables metallic plasmonic NPs to enhance the fluorescence intensity of fluorophores located near them, which is named metal-enhanced fluorescence (MEF) [146]. MEF is sensitive to the distance between metal and fluorophore, providing a way for constructing fluorescent biosensors. The other extensively exploited optical phenomenon of plasmonic NPs is surface-enhanced Raman scattering (SERS). When the molecules are adsorbed onto corrugated plasmonic NPs, the inelastic scattering of photons is greatly enhanced by factors up to 10^5^ or even larger [147]. As a result, a variation of Raman peak intensity can sensitively reflect the analyte-plasmonic NP connection.

These properties of plasmonic NPs are beneficial for improving the sensitivity and lowing LOD of biosensors. LSPR is a promising tool for producing colorimetric sensors. As mentioned above, the absorbance of plasmonic NPs varies with their morphology, size, and composition, causing a color change in solutions containing these NPs. Therefore, specific biomarkers can be quantitatively analyzed by detecting the color variation of solution samples induced by analyte-triggered aggregation or surface modification of plasmonic NPs (Fig. 14B). MEF occurs only when the distance between metallic plasmonic NPs and fluorophores is within 5–90 nm. This distance could be extended or shortened by the formation of analyte-plasmonic NP complexes, which in turn alters the fluorescence intensity of the plasmonic NPs/fluorophores system. This enables the development of highly-sensitive fluorescent biosensors for detecting biomarkers in bodily fluids. SERS biosensors usually assay analytes through two strategies: direct method and indirect approaches. The direct way is accomplished with the absorption of analyte onto plasmonic NPs, which results in a change in the Raman intensity.

This method requires both a close attachment between the analyte and the plasmonic NPs and a high Raman scattering cross-section of the analyte. For biomarkers with low or null Raman vibration modes, indirect detection is more suitable. The indirect detection involves the SERS spectrum shifts of a metabolite, reaction product, or reporter molecule (RM) that can reflect the concentration of the target biomarker [147–149].

**Fig. 15:**
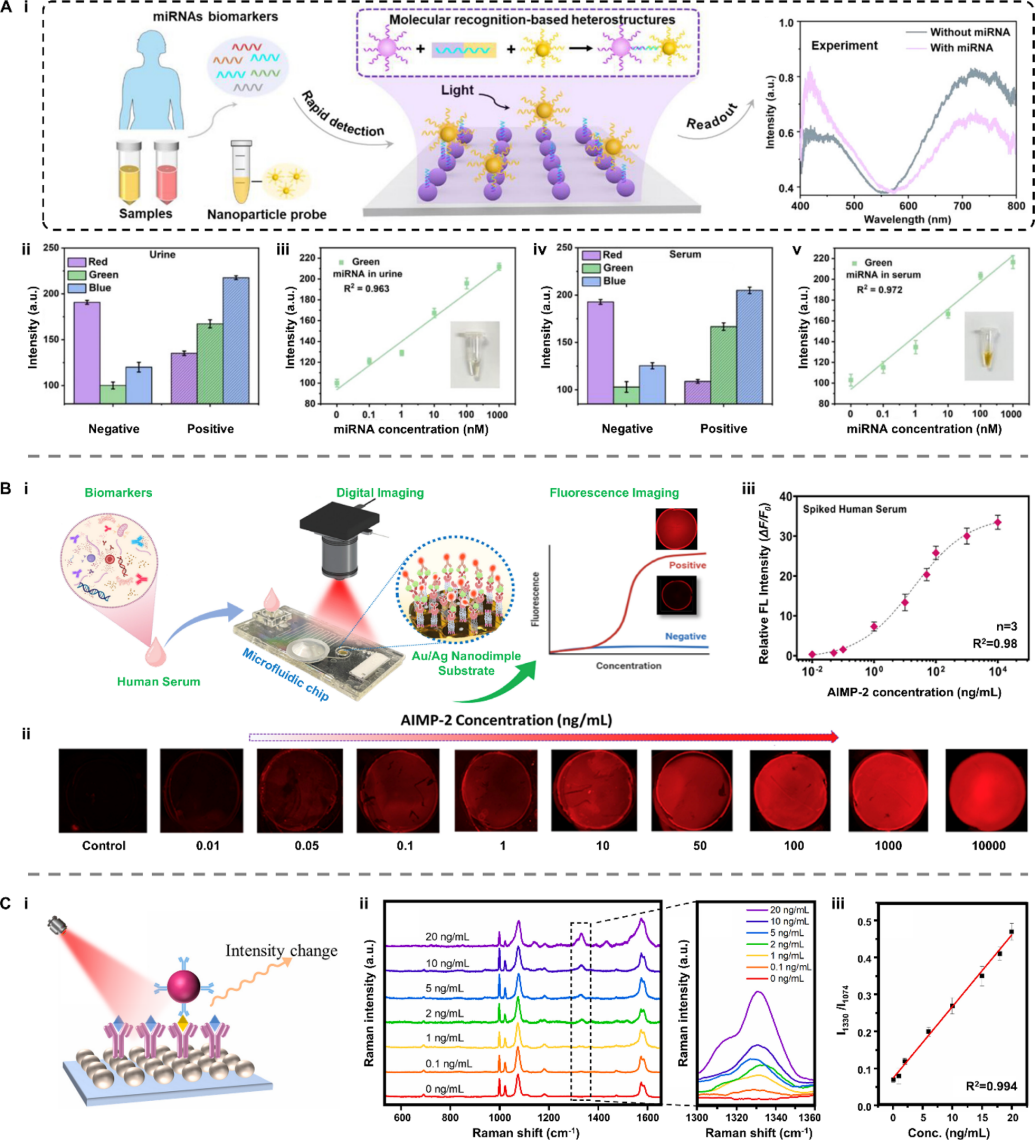
Applications of plasmonic nanoparticles in biosensing. **A** A portable colorimetric biosensor based on the LSPR mechanism for colorectal cancer-associated miRNAs assessment. **i** Schematic illustration of the working principle. The linear relationship between the miRNAs concentration and the imaging intensity in the green channel in **ii**, **iii** urine samples and **iv**, **v** serum samples, respectively. Reproduced with permission [150]. Copyright 2023, American Chemical Society. **B** A MEF-based biosensor for detecting the Parkinson’s disease biomarker, AIMP-2. **i** Schematic illustration of the working principle. **ii** The fluorescent images at different AIMP-2 concentrations. **iii** The nonlinear relationship between the logarithmic concentration of AIMP-2 and the relative fluorescent intensity. Reproduced with permission [148]. Copyright 2024, Elsevier. **C** A SERS-based biosensor using Ag NPs immunocolloidal probes for quantitively detecting f-PSA. **i** Schematic illustration of the working principle and **ii** feasibility of the SERS-based biosensor. **iii** The linear relationship between the f-PSA concentration and the SERS intensity ratio (*I*_*1330*_/*I*_*1074*_). Reproduced with permission [149]. Copyright 2023, Elsevier. AIMP-2: aminoacyl-tRNA synthetase complex interacting multi-functional protein 2; f-PSA: free prostate-specific antigen

Su et al*.* designed a portable colorimetric sensor based on the LSPR mechanism for detecting colorectal cancer-associated miRNAs [150]. In the presence of target miRNAs, two kinds of plasmonic NPs assemble into heterostructures, exhibiting obvious structure-mediated color changes according to LSPR (Fig. 15A-i). Urine and serum samples were tested to investigate the sensitivity and accuracy of this sensor. In the urine samples, the imaging color changed from red to blue (Fig. 15A-ii). The concentration of target miRNA showed a linear relationship with the imaging intensity in the green channel (R^2^ = 0.963) (Fig. 15Aiii). The results from the serum samples exhibited a similar effect (Fig. 15A-iv), and the intensity of these heterostructures in the green channel linearly increased with the concentration of the target miRNA (Fig. 15A-v).

As an example of MEF-based biosensor, Lee et al*.* proposed a point-of-care testing (POCT) system integrated with a capillary flow-driven microfluidic cartridge (CFMC) for the detection of the Parkinson’s disease biomarker, aminoacyl-tRNA synthetase complex interacting multi-functional protein 2 (AIMP-2) (Fig. 15B-i) [148]. The fluorescent intensity was significantly enhanced by the formation of a detection antibody (dAb)-target-capture antibody (cAb) sandwich structure, where target biomolecules first interacted with dissolved dAbs and then were captured by cAbs immobilized on the Au/Ag nanodimple (ND) substrate (Fig. 15B-ii). As a result, the biosensor signal (ΔF/F_o_) exhibited a nonlinear relationship with the logarithmic concentration at a range of 10^−2^–10^4^ ng mL^−1^ (R^2^ = 0.98) with a LOD of 0.004 ng mL^−1^.

Using the SERS mechanism, Zhao et al*.* developed a biosensor to quantitively detect free prostate-specific antigen (f-PSA) by the Raman intensity change of a reporter molecule (RM) [149]. Silver nanoparticles (Ag NPs) act as immunocolloidal probes, which were modified by the RM 5,5′-dithiothio (succinyl subunit-2-nitrobenzoate) (DSNB) (Fig. 15C-i). In the presence of f-PSA, the immunocolloidal probe was captured by the substrate, leading to a change in the characteristic peak of DSNB at 1330 cm^−1^ (Fig. 15C-ii). The SERS intensity ratio (*I*_*1330*_/*I*_*1074*_) was raised as the increase of the f-PSA concentration from 0.1 to 20 ng mL^−1^ linearly (R^2^ = 0.994) (Fig. 15C-iii).

In summary, light-responsive materials serve as essential components in constructing multifunctional and compact biosensors. Artificial enzyme mimics allow biosensors to have a shorter testing time and more detectable analytes by catalyzing diverse biochemical reactions. However, these redox processes mostly rely on the generation of reactive oxygen species (ROS), leading to poor specificity. Even though light-responsive QDs and MOFs are regarded as ideal materials for signal transducers in the photoelectrochemical biosensor, they still face challenges in complex liquid samples with multiple biomolecules such as whole blood. Plasmonic NPs can enhance fluorescence intensity by approximately 2- to 1000-fold compared to unmodified systems and reduce the LOD by 30- to 100-fold relative to conventional ELISA methods, depending on the specific biosensor design and target analyte [150, 151]. However, the generated output signals usually require further processes such as RGB analysis and Raman spectroscopy, posing a challenge in developing portable and affordable biosensors.

## Electro-responsive materials enabled biosensors

Electric manipulation is a favorable and mature technique that has been extensively exploited in various fields such as engineering manufacture, automation, and sensor due to its easy operation, fast response speed, and high programmability [152]. This technology also has promising application prospects in the biomedical area by offering high sensitivity and miniaturized systems. To further broaden its application in liquid biopsies, smart materials with the ability to respond to an electrical signal or electric field by changing their physical or chemical properties have been created. For example, electro-responsive materials such as piezoelectric materials and electro-active polymers (EAPs) deform themselves under electric stimuli, which are used for sensing pressure and strain [153, 154]. Other materials like conducting polymers (CPs) and functionalized MOFs act as an ideal matrix in electrochemical biosensors because of their properties of tunable conductivity and easy modification by functional groups [155]. Although applications of these electro-responsive materials in detecting biomarkers in bodily fluids have been reported by a large number of papers, they mainly serve as accessories for enhancing the sensitivity and selectivity of biosensors, which is out of this review’s focus. Therefore, this section provides a brief description, and a more detailed introduction can refer to previous reviews [156, 157].

### Piezoelectric materials

**Fig. 16:**
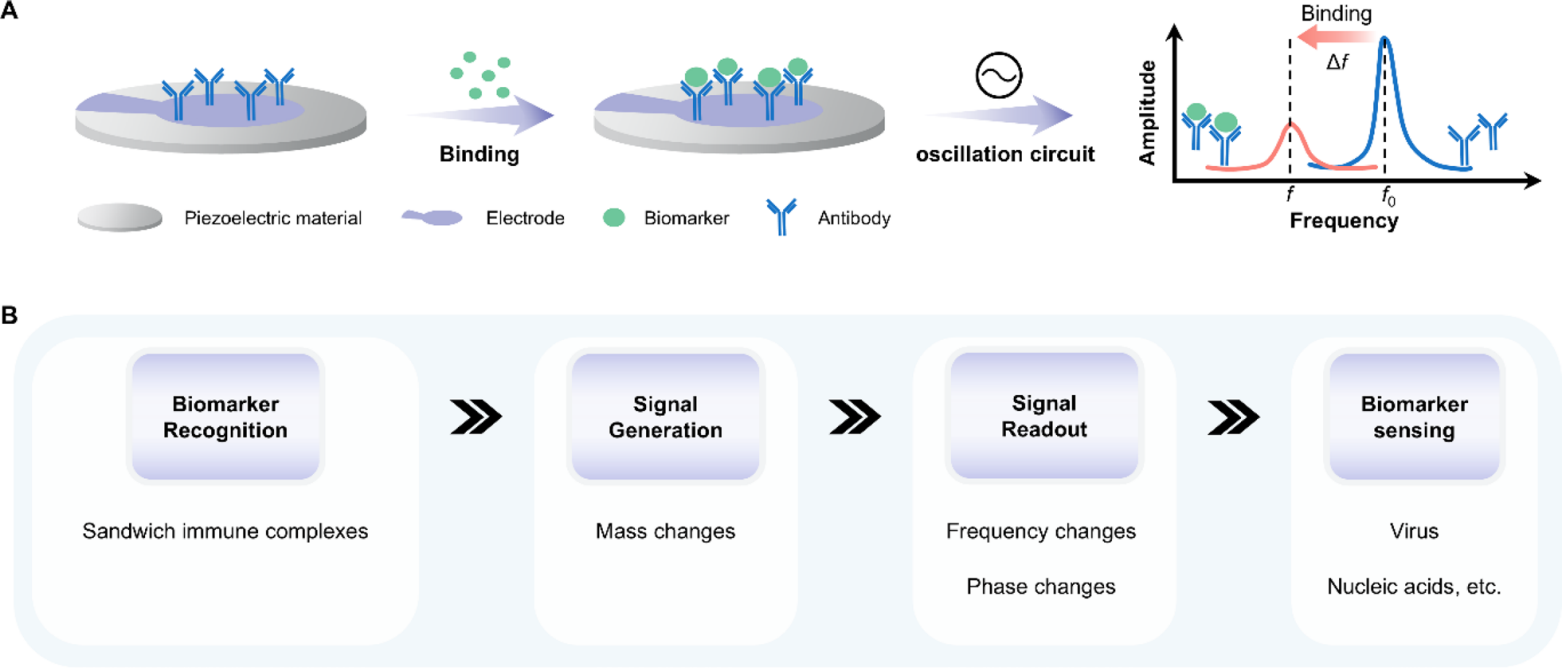
The working principle underlying electro-responsive piezoelectric materials’ applications in biosensing. **A** Schematic illustration of the decrease in the quartz oscillation frequency of piezoelectric materials when biomolecules are absorbed on their rigid surface. **B** The working principle underlying QCM in detecting biomarkers in bodily fluids. QCM: quartz crystal microbalance

Piezoelectric materials have the ability to transform mechanical stimulation into electrical signals, as discussed in Section “Piezoelectric materials”. Conversely, they can also act as electro-responsive materials [158]. Under an alternating current (AC) voltage, a piezoelectric material generates mechanical oscillation, producing an oscillating electric field. In 1959, Sauerbrey first discovered that the resonance frequency of a quartz-crystal oscillator changes as its surface mass. When biomolecules are absorbed on a rigid quartz-crystal surface, the mass accumulation on the quartz surface will cause a decrease in the quartz oscillation frequency in thickness shear mode (Fig. 16A). The Sauerbrey equation is given to define this Sauerbrey relationship:

1 $$\Delta m = -K \Delta F$$

where ∆*m* is the mass change [ng cm^−2^], ∆*F* is the frequency shift between the measured frequency and the fundamental resonance frequency [Hz], and *K* is a proportionality constant [ng cm^−2^ Hz^−1^]. Based on this Sauerbrey relation, Quartz Crystal Microbalance (QCM) has been extensively used over more than 50 years to quantitively analyze viruses and biomolecules (Fig. 16B). More detailed principles and comprehensive applications of QCM can be found elsewhere [159–161].

### Conductive polymers

**Fig. 17:**
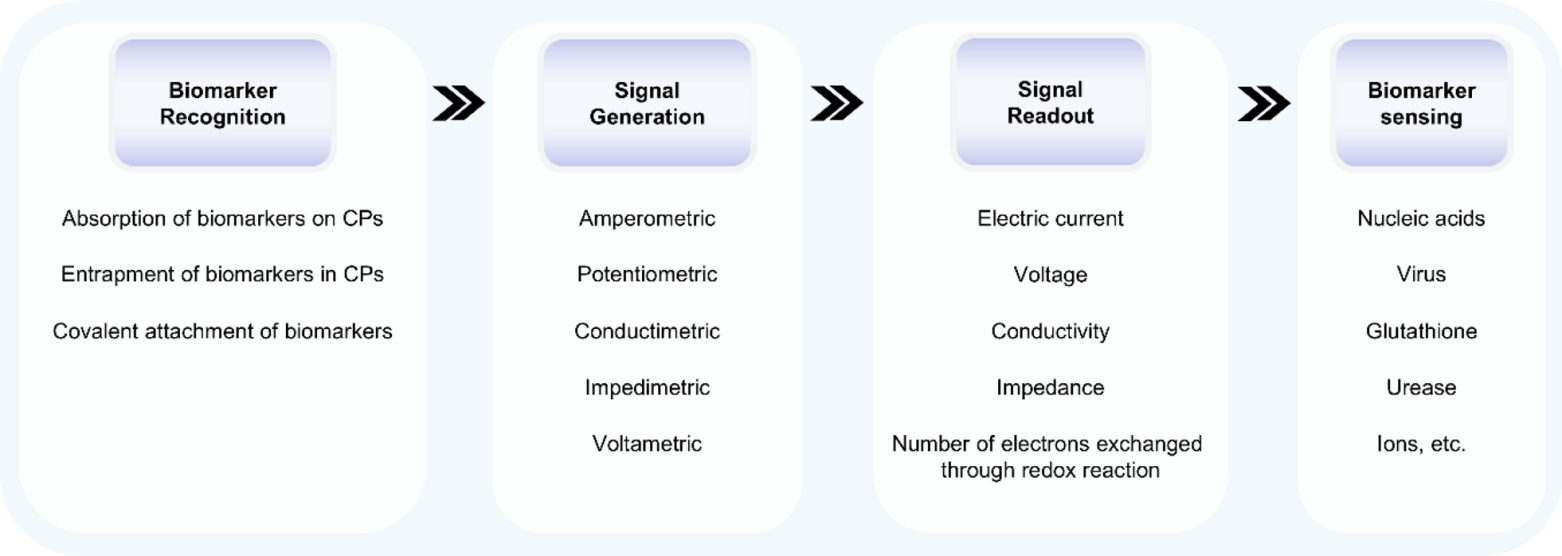
The working principle underlying conductive polymers’ applications in detecting biomarkers in bodily fluids

Conductive polymers (CPs) are an important category of smart materials that respond to an electrical field by changing shape and size [162]. This property allows them to be widely applied in the development of lightweight and flexible actuators, motors, and pressure/strain sensors [153, 154]. Additionally, CPs have drawn attention in the field of analytical chemistry due to their unique characteristics, including high conductivity, ease of modification by functional groups, and biocompatibility [163–167]. Therefore, CPs can serve as signal enhancers and converters in electrochemical biosensors for improving the sensitivity and selectivity of the biosensor while reducing the effect of interfering species (Fig. 17). When the target biomarker is captured by the bioreceptor immobilized on CPs-coated substrate, CPs convert the analyte-related information into electrochemical signals. This measurable signal can be a change in the value of the electric current, voltage, conductivity, impedance, or number of electrons exchanged through a redox reaction, resulting in the construction of amperometric [168], potentiometric [169], conductimetric [170], impedimetric [171], and voltammetric biosensors [172, 173]. Recent reviews have systematically summarized CPs’ preparation methods, classifications, and applications in biosensing [166, 174–176].

Electro-responsive materials-enabled biosensors show promising perspectives in commercialization and popularization. However, these biosensors also face challenges related to stability and sensitivity. First, the intrinsic properties of CPs make them susceptible to external factors such as pH, humidity, and temperature, which can alter their conductivity, surface properties, and mechanical integrity over time [177]. Second, biomolecules like proteins and cells in bodily fluids can nonspecifically adsorb onto the CPs'surface, an issue commonly referred to as biofouling, which compromises sensor functionality and reduces operational lifespan [178]. Third, while high sensitivity in electrochemical biosensors is often achieved by incorporating biological recognition elements such as enzymes, antibodies, or aptamers, poor binding stability and adhesion of these elements can result in reduced selectivity and increased signal noise. Due to the above factors, the validated operational period during which the biosensor maintains accuracy and stability is typically restricted to around two months [166]. Therefore, further research should prioritize the design and development of highly stable and sensitive receptor layers for both the QCM and electrochemical biosensors.

## Magnetic-responsive materials enabled biosensors

The magnetic control method is a fast-growing field due to its biocompatible, non-invasive, and high-throughput properties. This trend highlights magnetic-responsive materials, which deform, move, or generate heat upon exposure to a magnetic field. Magnetic-responsive materials can be single-component like magnetic nanoparticles (MNPs) made from pure superparamagnetic iron oxide, or multi-component, such as the composite polymer materials doped with MNPs or the MNPs coated with biomaterials. These smart materials show promising applications in developing wireless actuators [179], remote-manipulation robots [180], and magnetocaloric materials [181]. Magnetic-responsive materials are also prominent in constructing biosensors used for detecting biomarkers in bodily fluids. Their superparamagnetic properties make them highly effective for sample enrichment and separation, while their large surface area and high enzyme-mimicking activity enable significant signal amplification [182, 183]. In this section, we exclusively discuss the magnetic-responsive materials that convert biomarker-related information into readable signals in biosensors as detecting reporters.

### Magnetic nanoparticles

**Fig. 18:**
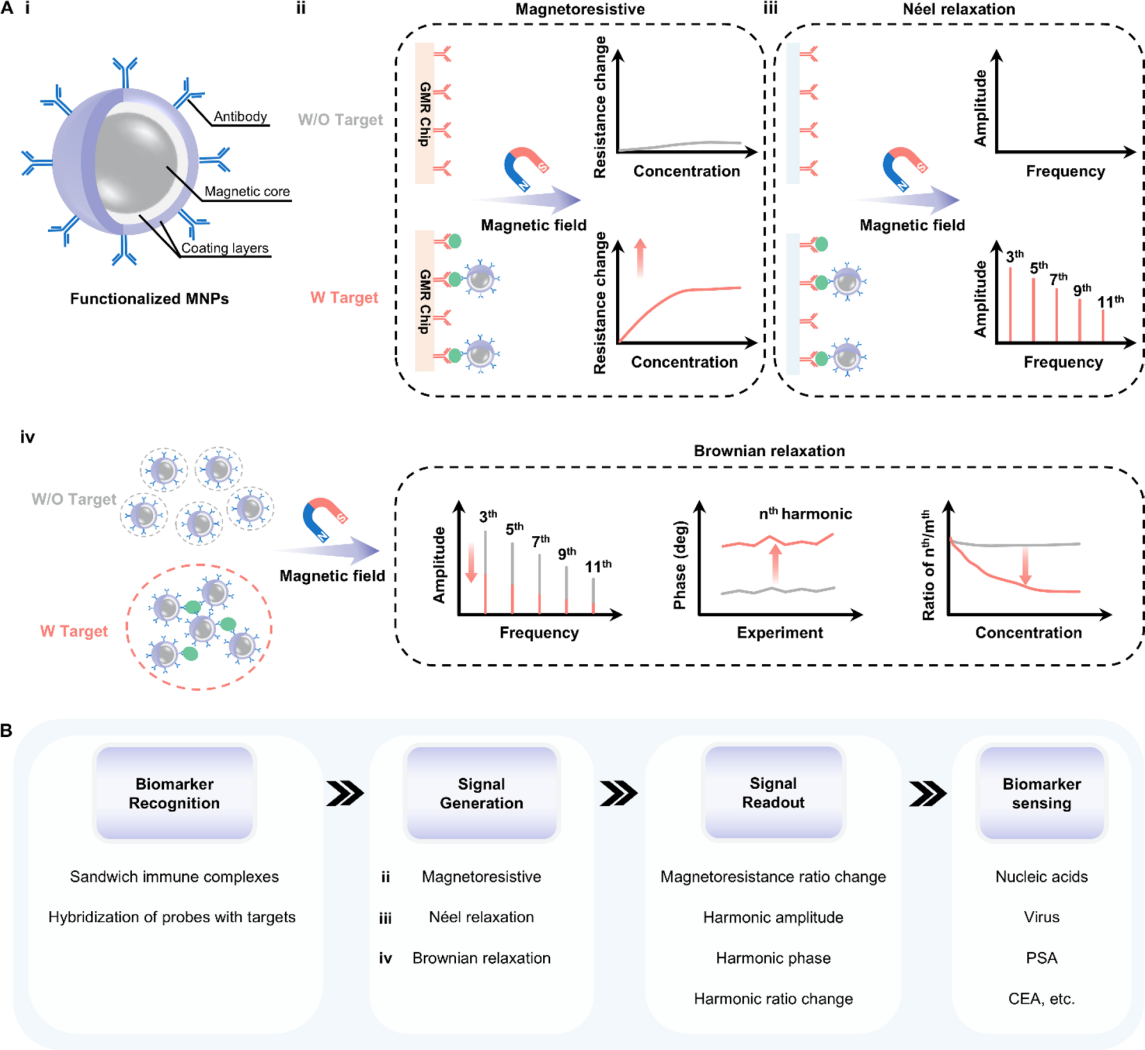
Representative type of MNPs and the working principles when applied in biosensors. **A**-**i** Schematic illustration of functionalized MNPs. MNPs-enabled biosensors based on the **ii** magnetoresistive property, **iii** Néel relaxation and **iv** Brownian relaxation. **B** The working principle underlying MNPs’ applications in detecting biomarkers in bodily fluids. MNPs: magnetic nanoparticles; PSA: prostate-specific antigen; CEA: carcinoembryonic antigen

MNPs can be remotely manipulated by an external magnetic field, which are usually made from materials with high saturation magnetizations, such as pure metals, ferrites and iron oxide. These NPs with a size ranging from 1 to 100 nm exhibit high surface-area-to-volume ratio and size-dependent physicochemical properties, endowing MNPs with unique magnetic and electrical properties [184]. To satisfy the requirement in the biomedical context, MNPs are further coated with biomaterials to develop their biocompatibility, colloidal stability and target selectivity (Fig. 18A-i). These functionalized MNPs have been used in magnetoresistive (MR) biosensors as detecting probes (Fig. 18A-ii). Under an external magnetic field, MNPs captured on the surface of a giant magnetoresistance (GMR) chip would generate a stray field, leading to a change in the resistance by altering electron tunneling [184–186].

MNPs-based magnetic particle spectroscopy (MPS) biosensors have been a fast-growing field in recent years. In a sinusoidal magnetic field, MNPs exhibit a dynamic magnetization response and produce a non-linear magnetization curve, which can be converted into MPS spectrum featuring higher odd harmonic amplitudes and phases after Fourier transformation [187, 188]. This magnetization response is realized by both Néel and Brownian relaxation processes of MNPs. Néel process is the internal flipping of the magnetic moment inside a stational MNP. In contrast, the Brownian process is the physical rotation of the MNP's magnetic moment along with its hydrodynamic shell outside. Therefore, a change in MNP's hydrodynamic size can dramatically influence the Brownian relaxation time, resulting in a variation of harmonic amplitude and phase lag on the MPS spectrum. This different rotational behavior of MNPs under the external magnetic field gives the two processes distinct roles in MPS-based applications [189–191]. The dominant relaxation of these two processes directly depends on many factors such as temperature, viscosity, and hydrodynamic size. In general, MNPs with magnetic core sizes smaller than 20 nm are Néel relaxation mechanism-dominated [192]. Due to their small size and unique rotational property, an increase in the hydrodynamic size of the MNPs through biomolecule-induced aggregation does not affect their MPS spectra. Therefore, the Néel relaxation-based MPS biosensor, also termed a surface-based MPS biosensor, needs the help of a sandwich bioassay technique to capture MNPs for quantifying analytes (Fig. 18A-iii). When magnetic core size increases above 20 nm, MNPs undergo Brownian relaxation processes. This movement is hydrodynamic volume-dependent. In the presence of target biomarkers, therefore, the hydrodynamic sizes of MNPs are increased by the analyte-induced aggregation, resulting in a rise in the relaxation time. Based on this strategy, the volumetric-MPS biosensor is developed to detect target biomarkers by detectable changes in harmonic amplitudes, phases, and harmonic ratios (Fig. 18A-iv).

**Fig. 19:**
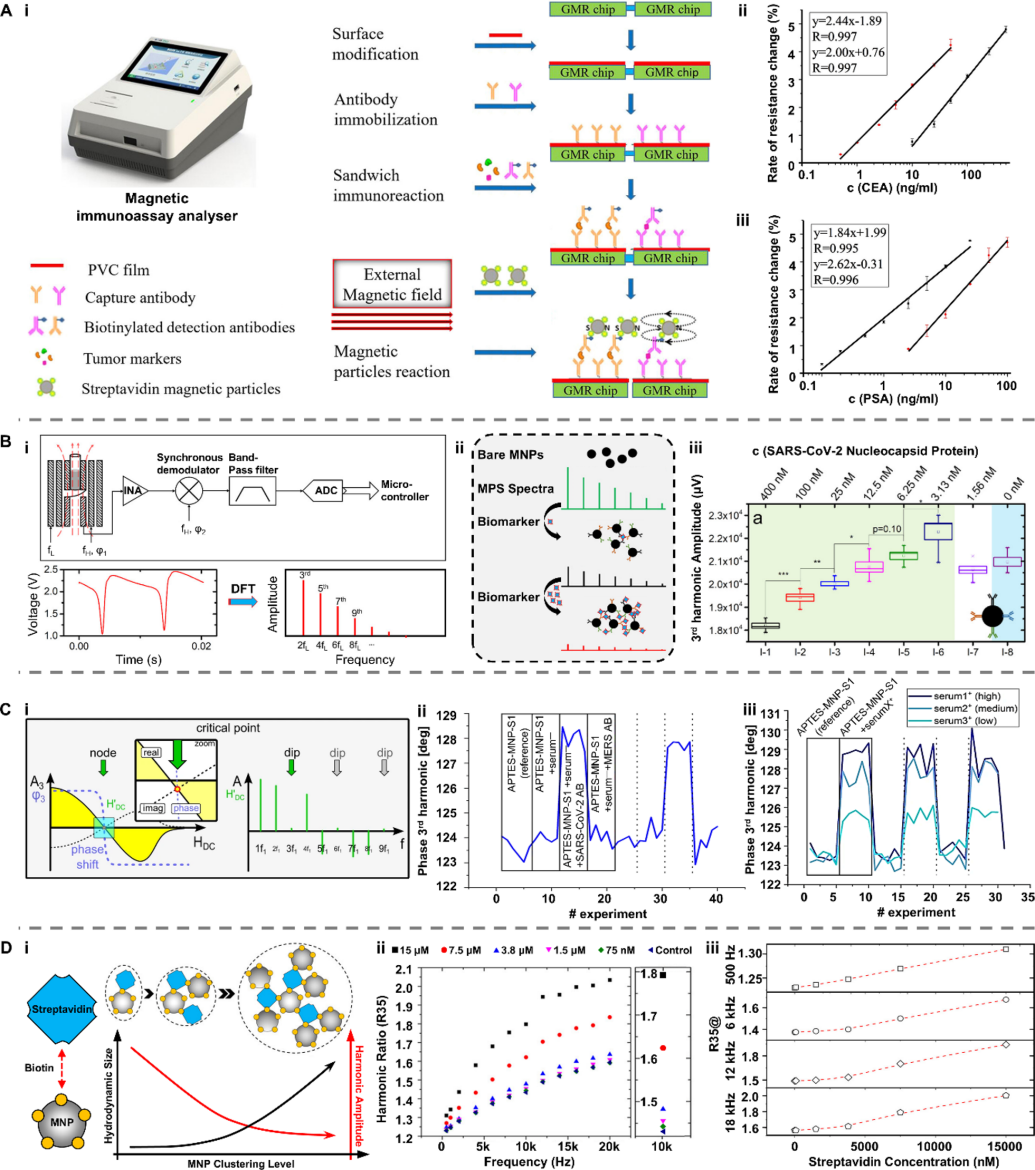
Applications of MNPs in biosensing. **A** A magnetic immunoassay analyzer based on the magnetoresistive mechanism for simultaneously detecting twelve tumor markers. **i** Schematic illustration of the setup and working principle of the GMR immunoassay biosensor. **ii** The linear relationship between the logarithm of CEA concentration and the resistance change using two different capture antibodies. **iii** The linear relationship between the logarithm of PSA concentration and the resistance change using two different capture antibodies. Reproduced with permission [198]. Copyright 2019, Elsevier. **B** A wash-free volumetric-MPS biosensor for quantifying SARS-CoV-2 spike and nucleocapsid proteins. Schematic illustration of **i** the setup and **ii** working principle of the GMR immunoassay biosensor. **iii** The 3rd harmonics monotonically increase as the concentration of nucleocapsid protein decreases (highlighted green areas). Reproduced with permission [199]. Copyright 2021, American Chemical Society. **C** A Critical Offset Magnetic PArticle SpectroScopy (COMPASS) for sensitive point-of-care diagnostics. Schematic illustration of **i** the working principle and **ii** feasibility of the COMPASS. **iii** Results for three different blood sera. Reproduced with permission [200]. Copyright 2022, Springer Nature. **D** A Brownian relaxation-based MPS biosensor for detecting streptavidin. Schematic illustration of **i** the working principle; and **ii** feasibility of the proposed MPS biosensor. **iii** The relationship between streptavidin concentration and the ratio of the 3rd to the 5th harmonics (R35) at different driven frequencies. Reproduced with permission [195]. Copyright 2019, American Chemical Society. MNPs: magnetic nanoparticles; GMR: giant magnetoresistance; CEA: carcinoembryonic antigen; PSA: prostate-specific antigen; MPS: MNPs-based magnetic particle spectroscopy

The diversity of MNPs-based biosensors enables the detection of various biomolecules in biofluidic samples (Fig. 18B) [189, 193–197]. For instance, Gao et al*.* developed a GMR immunoassay biosensor with the ability to simultaneously detect twelve tumor markers within 15 min (Fig. 19A-i) [198]. Using MNPs modified with two capture antibodies, the immunosensor’s resistance change had a linear response with the logarithm of analyte concentrations in the range of 0.5–500 ng mL^−1^ for carcinoembryonic antigen (CEA) (Fig. 19A-ii), and 0.1–100 ng mL^−1^ for total prostate-specific antigen (PSA) (Fig. 19A-iii).

As an example of the volumetric-MPS biosensor, Wang et al*.* designed a one-step and wash-free diagnostic platform for quantifying SARS-CoV-2 spike and nucleocapsid proteins in liquid phase [199]. The signal from pick-up coils was amplified by a high-precision instrumentation amplifier (INA828) and then processed by a one-stage lock-in implementation, which consisted of a synchronous demodulator followed by bandpass filtering. This one-stage lock-in implementation enables to improve the detection sensitivity by removing the feedthrough signals corresponding to the excitation magnetic field frequencies and only recording the dynamic magnetic responses of MNPs. Thus, the filtered voltage signal from the lock-in implementation was converted into MPS spectra after the discrete Fourier transform (Fig. 19B-i). Polyclonal antibodies (pAbs)-modified MNPs were specifically bound to the target protein molecule, leading to a change in the harmonic amplitudes based on the Brownian relaxation mechanism (Fig. 19B-ii). The 3rd harmonics monotonically increased as the concentration of nucleocapsid protein decreased from 400 (32 pM) to 3.13 nM (500 fM) (highlighted green areas in Fig. 19B-iii).

As an alternative technique to enhance the sensitivity of MPS biosensors, Behr et al*.* combined a strong time-varying excitation field *H*_AC_ with a strong constant offset magnetic field *H*_DC_, named Critical Offset Magnetic PArticle SpectroScopy (COMPASS) [200]. In the presence of an *H*_DC_, the magnetization response *M*(*t*) becomes asymmetric. When *H*_DC_ < *H*_AC_, spectral component *A*_n_(*H*_DC_) that is the amplitude of higher harmonic *n* at offset magnetic fields *H*_DC_ shows several nodes and the corresponding phase plot *φ*_n_(*H*_DC_) of the harmonic signal exhibited a steep slope of the phase near such nodes or “dips” (Fig. 19C-i). Therefore, minimal changes in MNPs'mobility caused by variations in their hydrodynamic diameter led to a strong detectable phase difference (Fig. 19C-ii, iii). This COMPASS device achieved a LOD of ~ 2 ng mL^−1^ in detecting SARS-CoV-2-S1 IgG antibody, which was comparable with the gold-standard methods ELISA and flow cytometry. Although the direct correlation between specific harmonic amplitudes and target biomarker concentrations offers a straightforward quantification approach, it is susceptible to bias due to variations in MNP quantities across samples, especially in low-concentration detection scenarios [195]. To overcome this challenge, Wang et al. introduced MNP quantity-independent metrics and demonstrated the practicality of MNP Brownian relaxation-based MPS for biosensing using a streptavidin–biotin binding system (Fig. 19D-i) [195]. In this approach, the ratio of the 3rd to the 5th harmonics (R35) at different drive field frequencies was employed as the MNP quantity-independent metric for characterizing biomarker concentrations. The R35 ratio increased proportionally with streptavidin concentration (Fig. 19D-ii), a trend consistently observed across various drive frequencies (Fig. 19D-iii). This work not only improves the accuracy of MPS biosensors by employing a metric independent of MNP quantity but also offers an efficient alternative for conducting MPS bioassay measurements, as it does not require the full screening of the drive field frequencies.

In summary, magnetic-responsive materials-enabled biosensors provide a biocompatible, sensitive, and feasible platform to detect and analyze biomarkers in bodily fluids. However, the relaxation time is influenced by many factors including the shape and size of MNPs. Thus, for obtaining biosensors with better accuracy, MNP synthesis methods should be improved to prepare nanoparticles with higher saturation magnetizations and better size uniformity. Besides, multiple and repeated washing is required to improve the accuracy of MNPs-based MPS biosensors, and the functionality of this kind of biosensor is not yet fully satisfied. For future clinical and point-of-care applications, it is important to simplify the testing process, narrow down testing time, and improve functionality.

## Thermo-responsive materials enabled biosensors

Biochemical reactions always accompany a change in energy, usually in the form of heat. Temperature thus is regarded as a critical standard to quantify biomolecules involved in biochemical reactions. Thermo-responsive materials satisfy the need to construct thermal biosensors by visualizing temperature variation. Under a temperature stimulus, smart materials undergo a discontinuous phase transition or morphological change, where thermo-responsive polymers and shape memory alloys are common examples [201, 202]. Despite the different components and working principles, these kinds of materials can be mainly classified into two types based on their temperature-responsive behaviors: lower critical solution temperature (LCST) and upper critical solution temperature (UCST) [203]. In addition, thermochromic materials display different colors at different temperatures, which can act as signal transducers to report temperature variations in a readable manner. Therefore, this section focuses on thermochromic materials that are utilized to develop thermal biosensors for detecting biomarkers in bodily fluids.

### Thermochromic materials

**Fig. 20:**
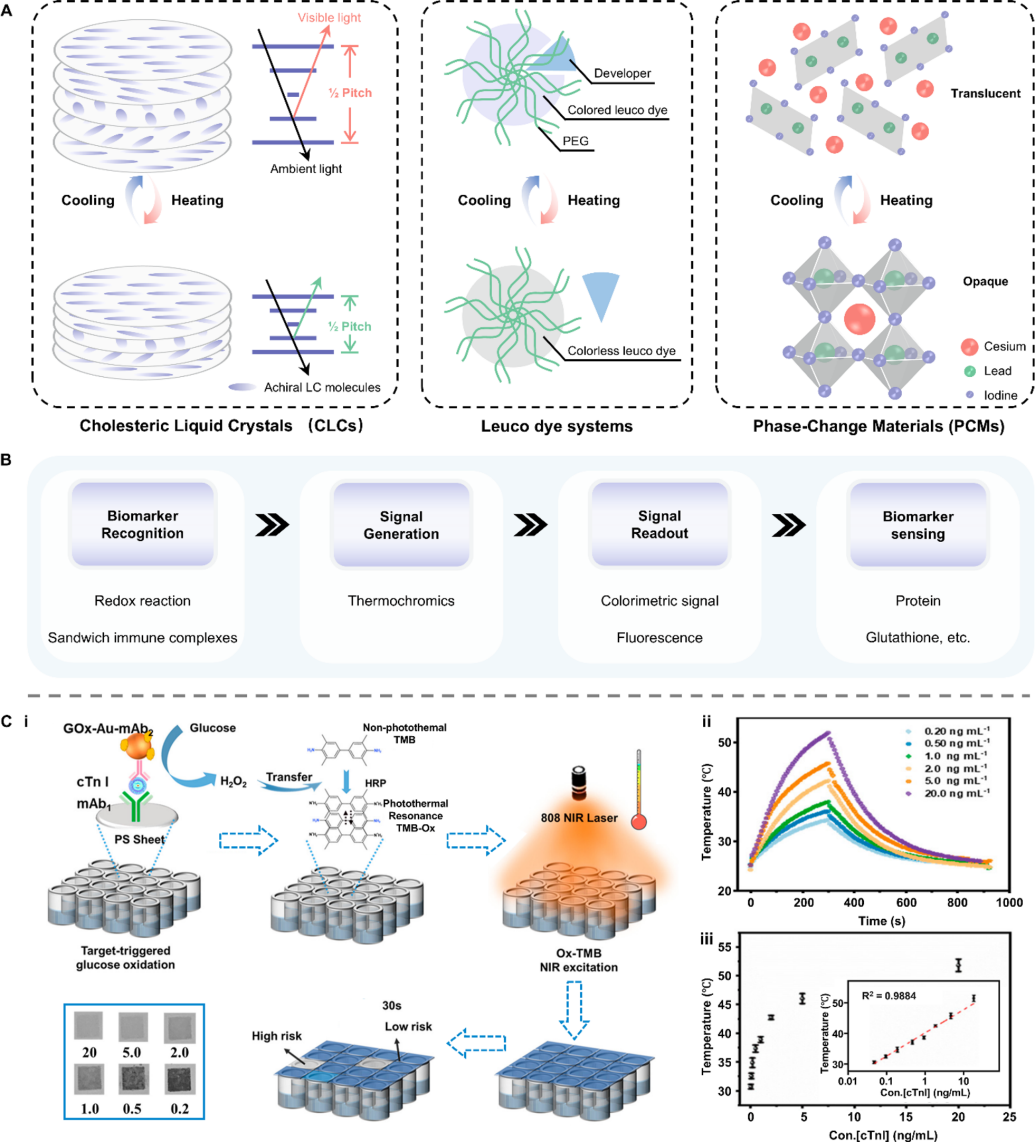
Classifications, responsive mechanisms and typical applications of thermochromic materials. **A** Schematic illustration of thermochromic mechanisms of three thermo-responsive materials including cholesteric liquid crystals (CLCs), leuco dye systems and phase-change materials (PCMs). **B** The working principle underlying thermochromic materials’ applications in detecting biomarkers in bodily fluids. **C** A thermochromic paper-based photothermal biosensor for rapid screening of acute myocardial infarction. Schematic illustration of **i** the working principle and **ii** feasibility of the photothermal biosensor. **iii** The linear relationship between the concentration of cTnI protein and the maximum temperature. cTnI protein: cardiac troponin I protein. Reproduced with permission [211]. Copyright 2022, American Chemical Society

Thermochromic materials switch visual color as a response to a thermal stimulus and are mainly classified into three types based on their temperature-responsive mechanism: cholesteric liquid crystals (CLCs), leuco dye systems, and phase-change materials (PCMs). CLCs feature a helical structure that follows Bragg’s law, i.e. *λ* = *nP* cos*θ*, where *n* refers to the average refractive index and *θ* is the angle of observation (Fig. 20A-i). The pitch (period) *p* can be adjusted by temperature, with a range of a few hundred nanometers. Based on Bragg’s law, a thermal stimulus will change the *p* of CLCs followed by a shift in reflection peak, resulting in a variation in visual appearance. The development of CLCs has made significant progress recently, and CLCs have been designed with the ability to display colors across the entire visible spectrum under thermal modulation [204, 205]. The leuco dye system consists of a leuco dye connected with a color developer by a solvent such as poly(ethylene glycol) (PEG) (Fig. 20A-ii). Upon heating, the leuco dye–developer–solvent system achieves a transformation from a colored solid state to a colorless molten state due to the absence of the developer as solvents melt [206]. Alternatively, PCMs exhibit thermochromic phenomena through phase transformation (Fig. 20A-iii) [207].

Inspired by these principles, heat accumulation generated in a redox reaction can be converted into measurable signals, which are further used to detect biomolecules (Fig. 20B) [208–210]. For instance, Tang et al. developed a photothermal biosensor using thermochromic paper to diagnose acute myocardial infarction (Fig. 20C-i) [211]. Crystalline violet lactone (CVL) featuring color change response at around 45 °C served as signal reporter. In the presence of target cardiac troponin I (cTnI) protein, a cascade enzyme amplification reaction was triggered to generate oxidized 3,3’,5,5’-Tetramethylbenzidine (TMBox), which produced a certain amount of heat under an 808 nm NIR laser irradiation (Fig. 20C-ii). As a result, the color of the thermochromic paper was changed and used to report the concentration of cTnI protein. The target concentration ranging from 0.05 to 20 ng mL^–1^ showed a good linear correlation with the maximum temperature (*R*^2^ = 0.9884) (Fig. 20C-iii). The LOD was 0.021 ng mL^−1^.

Although thermo-responsive materials have been well applied in various fields, their biomedical application is still underexplored. Current thermo-responsive materials-enabled biosensors are mainly based on the heat release of a biochemistry reaction, which has limited specificity. In addition, the testing time and detecting accuracy still need to be improved to meet the requirement of practical usage.

## Ion-responsive materials enabled biosensors

Our bodily fluids contain a large variety of ions and electrolytes, which play an important role in maintaining bodily and cellular functions [212]. On this basis, ion-responsive materials with biocompatibility are sought-after to construct biosensors for health monitoring and disease diagnosis. Under different ionic stimuli, ion-responsive materials mainly change their physical properties (e.g., stiffness, viscoelasticity, solubility, etc.) [213], or lead to a variation in reflecting color/fluorescence, a subclass particularly favored in biosensing applications. This section discusses two types of fluorescent materials based on different stimulated ions: pH-responsive chromophores and ion-responsive aggregation-induced emission luminogens (AIEgens).

### pH-responsive chromophores and fluorophores

**Fig. 21:**
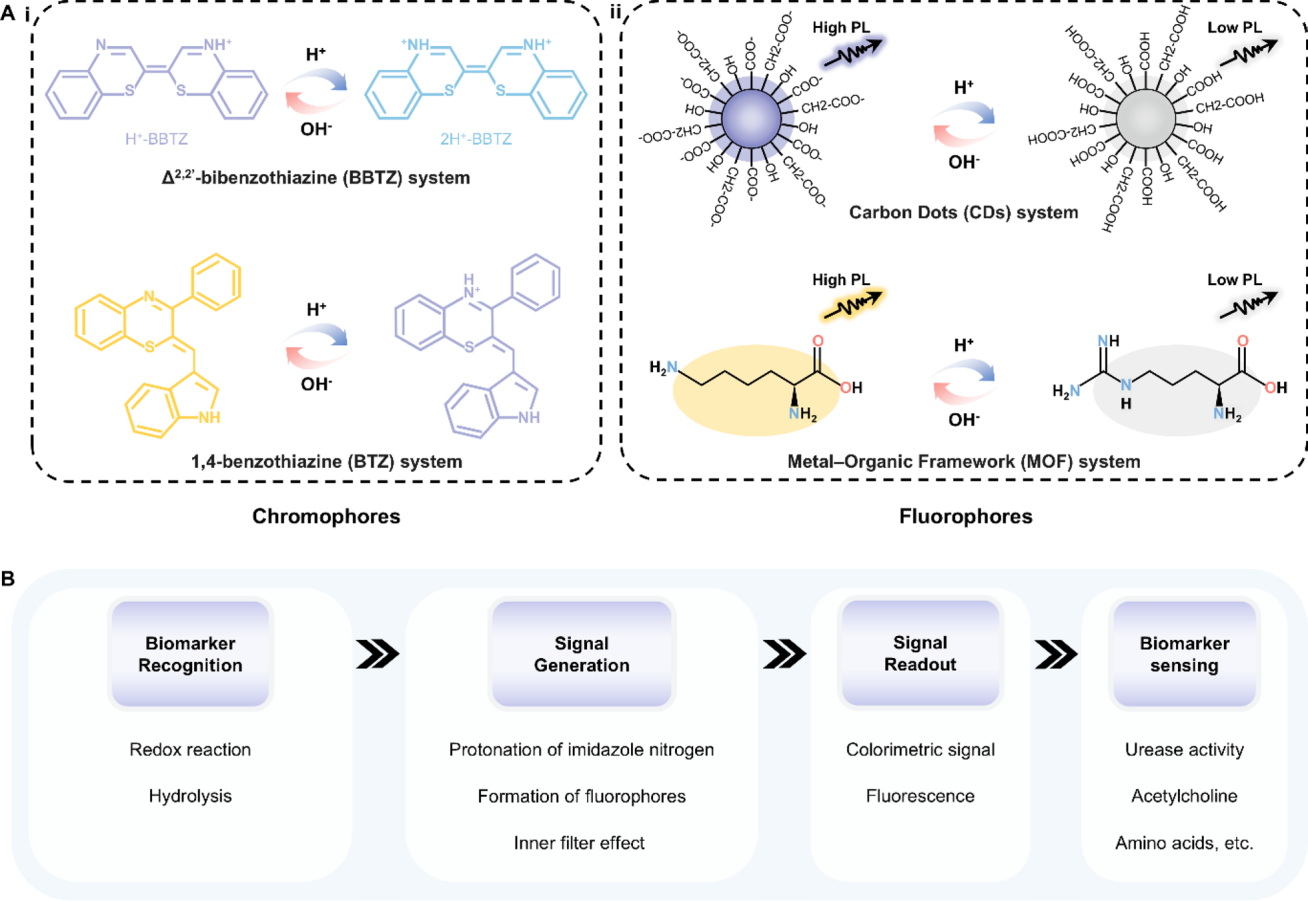
Representative type of pH-responsive chromophores and fluorescent materials and their working principles when applied in biosensors. **A** Schematic illustration of pH-responsive **i** chromophores and **ii** fluorophores. **B** The working principle underlying MNPs’ applications in detecting biomarkers in bodily fluids. PL: photoluminescence

Many biochemical reactions via enzyme proteins in metabolism will change the pH of bodily fluid. For example, urea can be specifically hydrolyzed by urease to yield carbon dioxide and ammonia, leading to an obvious increase in the pH value [214]. This phenomenon provides an indirect strategy to detect enzyme activity and metabolites by quantifying pH. Therefore, great efforts have been exerted to develop versatile pH-responsive materials. For instance, based on the dopaquinone-cysteine coupling reaction, the Δ2,2′-bibenzothiazine (BBTZ) system with robust acidichromism is designed, which shows a transformation from a violet cationic form (H^+^-BBTZ) to a deep blue dication (2H^+^-BBTZ) with pH increase (Fig. 21A-i). Additionally, H^+^ is proven to be able to quench the fluorescence of nitrogen-doped QDs or MOFs by protonating imidazole nitrogen, allowing them to be used as promising fluorescent reporters (Fig. 21A-ii). The detailed and latest development of pH-responsive chromophores and fluorescent materials can refer to previous reviews [215, 216].

**Fig. 22:**
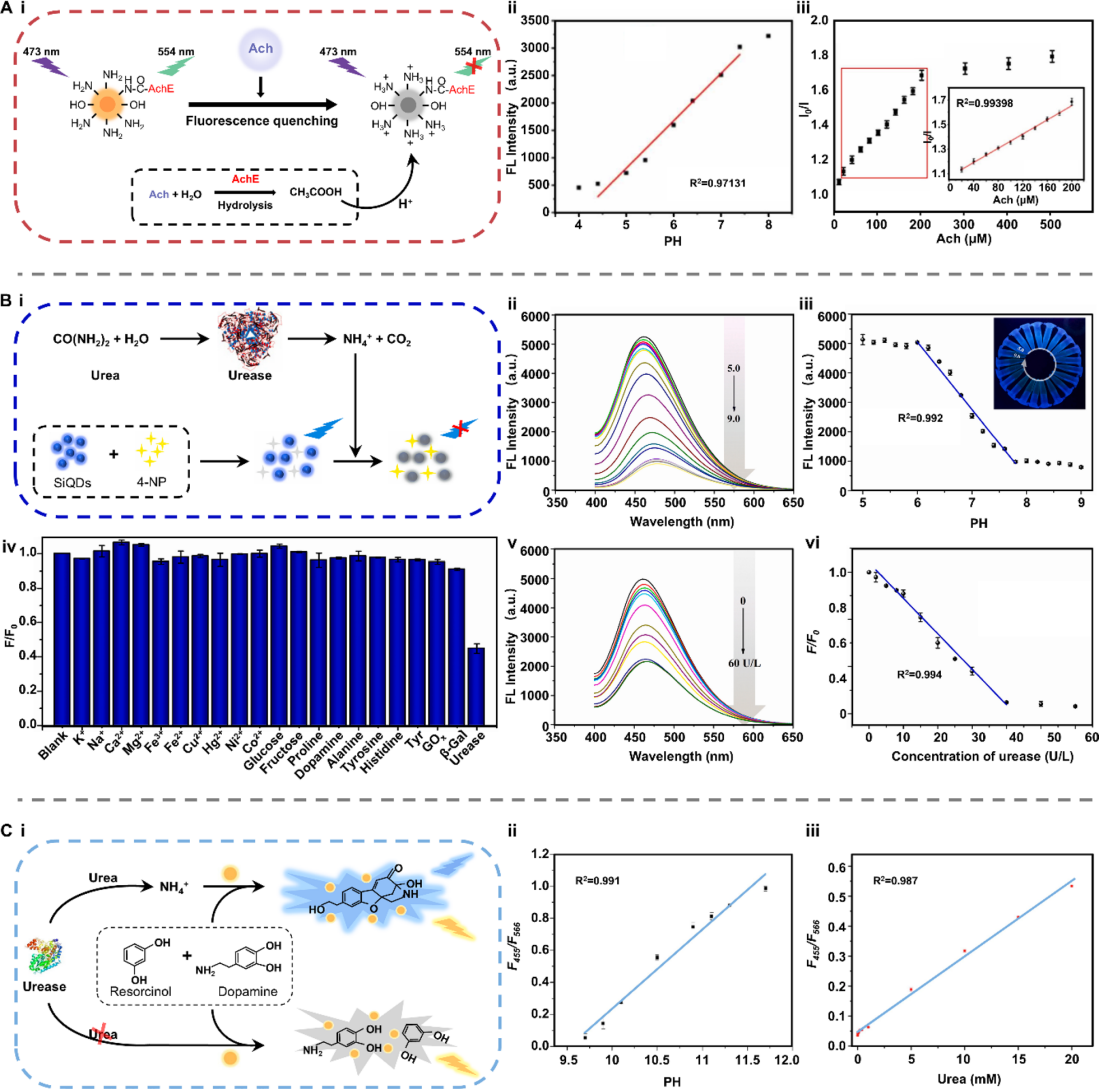
Applications of pH-responsive chromophores and fluorescent materials in biosensing. **A** An optical fibre biosensor for acetylcholine detection based on pH sensitive fluorescent CQDs. Schematic illustration of **i** the working principle and **ii** feasibility. **iii** The linear relationship between the AchCl concentration and the I_0_/I value, defined as the fluorescence intensity of the fiber biosensor absence and presence of AchCl in test solution. Reproduced with permission [221]. Copyright 2022, Elsevier. **B** A pH-responsive fluorometric biosensor based on SiQDs and 4-NP for urease activity detection. **i** Schematic illustration of the working principle. **ii**, **iii** The linear relationship between the pH and the fluorescence intensity. **iv** Specificity of the proposed fluorometric biosensor. **v**, **vi** The linear relationship between the urease concentration and the fluorescence intensity. Reproduced with permission [222]. Copyright 2022, Elsevier. **C** A pH-responsive ratiometric fluorescence system based on AIZS QDs and Aza for urea detection. **i** Schematic illustration of the working principle. **ii** The linear relationship between the pH and the ratio of the fluorescence intensity *F*_*455*_/*F*_*566*_. **iii** The linear relationship between the urea concentration and ratio of the fluorescence intensity *F*_*455*_/*F*_*566*_. Reproduced with permission [223]. Copyright 2022, Elsevier. CQDs: carbon quantum dots; Ach: acetylcholine; AchE: acetylcholinesterase; AchCl: acetylcholine chloride; SiQDs: silicon quantum dots; 4-NP: 4-nitrophenol; AIZS QDs: Zn doped AgInS_2_ quantum dots; Aza: azamonardine

On this basis, these smart materials can serve as signal converters in biosensors with the help of pH-involved biochemical reactions (Fig. 21B) [217–220]. Using nitrogen-doped fluorescent carbon quantum dots (N-CQDs), Li et al*.* proposed an optical fiber biosensor for acetylcholine (Ach) detection [221]. Ach generated acetic acid and choline after being hydrolyzed by acetylcholinesterase (AchE), which decreased the system pH value. As a result, the fluorescence of N-CQDs was diminished due to the protonation and aggregation of N-CQDs in acid solutions (Fig. 22A-i). The FL intensity of the AchE/CQDs/CA system linearly decreased as the decrease of pH in the range of 5.4–7.4 (R^2^ = 0.97131) (Fig. 22A-ii). Finally, the acetylcholine chloride (AchCl) concentration ranging from 20 µM L^−1^ to 200 µM L^−1^ exhibited a linear relationship with the I_0_/I value (the fluorescence intensity of the fiber biosensor absence and presence of AchCl in test solution) with a LOD of 16.28 µM L^−1^ (R^2^ = 0.99398) (Fig. 22A-iii).

Similarly, Su et al*.* developed a fluorometric system for urease activity detection (Fig. 22B-i) [222]. Urease catalyzed the hydrolysis of urea, increasing the alkalinity of the urine. Such a pH increase enhanced the absorption of 4-nitrophenol (4-NP) at 400 nm, followed by the fluorescence quenching of silicon quantum dots (SiQDs) at 460 nm due to the overlap of absorption of 4-NP with the fluorescence excitation spectrum of SiQDs (also termed as inner filter effect) (Fig. 22B-ii). The FL intensity of the SiQDs/4-NP sensing platform was linearly dependent on the pH value within the range of pH 6.0–7.8 (R^2^ = 0.992) with a precision of 0.2 pH unit (Fig. 22B-iii). These assay platforms possessed high selectivity (Fig. 22B-iv). In the detection of urease, the linear behavior was observed between the assay system pH and the F/F_0_ (FL intensities of SiQDs/4-NP (pH 5.0)/urea assay platform with and without urease) at the range of 2–40 U L^−1^, R^2^ = 0.994 (Fig. 22B-v,vi). The LOD was calculated to be 1.67 U L^−1^.

More recently, Su et al*.* developed a pH-responsive ratiometric fluorescence system for urea activity determination by combining pH-sensitive azamonardine (Aza) and pH-insensitive Zn doped AgInS_2_QDs (AIZS QDs) [223]. When the pH raised induced by the hydrolysis of urea, Aza was formed and its fluorescence at 455 nm was enhanced while the FL intensity of AIZS QDs at 566 nm remained unchanged. As a result, the ratio of the FL intensity (*F*_*455*_/*F*_*566*_) was utilized to detect urea (Fig. 22C-i). Results showed that this ratiometric fluorescence platform exhibited a linear response to pH values in the range of 9.7 to 11.7 at intervals of 0.2 (R^2^ = 0.991) (Fig. 22C-ii) and urea concentration ranging from 0.02 to 20 mM with the LOD of 0.0103 mM (R^2^ = 0.987) (Fig. 22C-iii).

### Ion-responsive aggregation-induced emission luminogens (AIEgens)

**Fig. 23:**
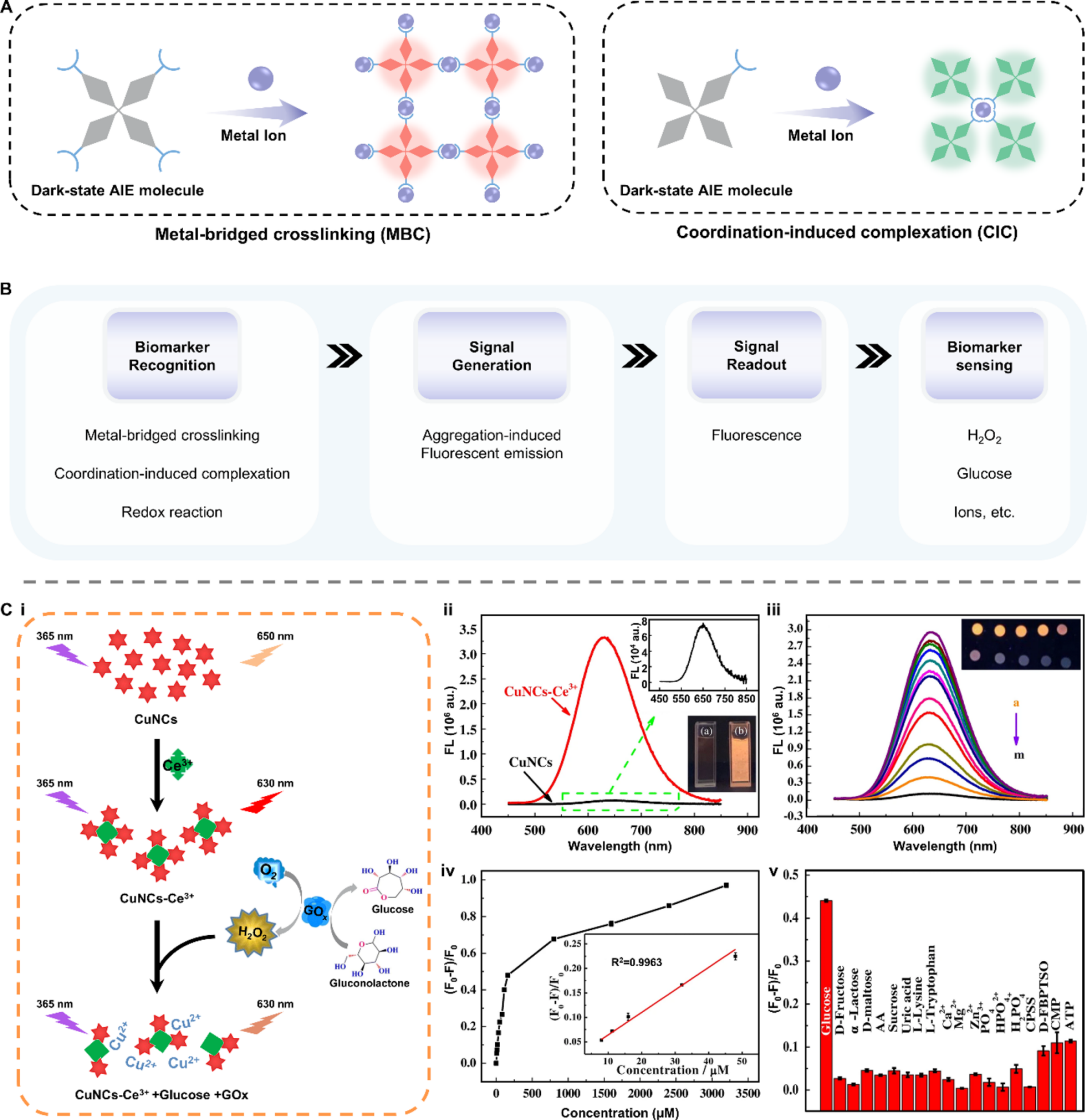
Responsive mechanism of ion-responsive AIEgens and their working principle when applied in biosensors. **A** Schematic illustration of two ion-responsive mechanisms including **i** metal-bridged crosslinking (MBC) and **ii** coordination-induced complexation (CIC). **B** The working principle underlying ion-responsive AIEgens’ applications in detecting biomarkers in bodily fluids. **C** A fluorescent assay of H_2_O_2_ and glucose based on a sensitive CuNCs-Ce^3+^ fluoroprobe. Schematic illustration of **i** the working principle and **ii** feasibility. **iii** Fluorescence spectra of CuNCs-Ce^3+^ with 160 μL of the different glucose concentrations. **a**–**m** 0 mM; 0.05 mM; 0.07 mM; 0.1 mM; 0.2 mM; 0.3 mM; 0.5 mM; 0.7 mM; 1 mM; 5 mM; 10 mM; 15 mM; 20 mM). **iv** The linear relationship between the glucose concentrations and the values of (*F*_0_–*F*)/*F*_0_. **v** The specificity of the proposed fluorescent assay. Reproduced with permission [235]. Copyright 2021, Springer Heidelberg. AIEgens: aggregation-induced emission luminogens; CuNCs-Ce^3+^: copper nanoclusters-Ce(III)

Traditional luminophores often exhibit weaker emission in the aggregated state because of the aggregation-caused quenching (ACQ) effect [224]. In contrast, aggregation-induced emission luminous (AIEgens) are highly emissive in concentrated solution or solid-state while emitting no light in the diluted solution state [224]. This phenomenon was first observed by Scheibe and Jelley independently in 1936 and officially coined by Ben Zhong Tang and co-workers in 2001 [225–227]. Since then, AIEgens gained increasing attention and ion-responsive AIEgens have been developed. In the presence of specific ions, dark-state AIE molecules are aggregated to restrict the intramolecular motions, which results in an increase in photoluminescence (PL) intensity by facilitating energy and charge transfer. The aggregation mechanisms behind the ion-responsive AIEgens vary from chemical reactions to self-assembly with target ions. Among these principles, metal-bridged crosslinking (MBC) and coordination-induced complexation (CIC) are the mostly exploited (Fig. 23A). The categories, synthesis method, and working principles of the ion-responsive AIEgens were systematically summarized in other reviews [228, 229].

The unique properties of AIEgens can be applied to construct turn-on or turn-off fluorescent biosensors for probing critical ions and metabolites in bodily fluids (Fig. 23B) [230–234]. For example, Wang et al. developed a fluorescent probe for the detection of H_2_O_2_ and glucose using glutathione-capped copper nanoclusters (CuNCs)-Ce(III) (CuNCs-Ce^3+^) mixing system (Fig. 23C-i) [235]. The FL intensity of CuNCs was significantly enhanced by the Ce^3+^ -triggered AIE process (Fig. 23C-ii) and specifically quenched by the addition of H_2_O_2_, which was attributed to oxidation of Cu^0^ or Cu^+^ in CuNCs to Cu^2+^. Based on this, the CuNCs-Ce^3+^ system was used to quantify glucose, which produced H_2_O_2_ under glucose oxidase (Go_x_) oxidization. The fluorescence (FL) intensity of the CuNCs-Ce^3+^/glucose/GOx system decreased gradually as the glucose concentration increased (Fig. 23C-iii). A linear relationship was observed between the glucose concentrations at a range of 8–48 μM and the values of (*F*_0_-*F*)/*F*_0_ (R^2^ = 0.9963) (Fig. 23C-iv). This turn-off fluorescent biosensor exhibited good selectivity for determining glucose with the LOD of 2.4 μM (Fig. 23C-v).

In summary, ion-responsive materials enable biosensors to detect ions in a direct way and other target biomolecules in an indirect way. Moving forward, it is expected to see more optimal biochemical and physical methods for preparing sensitive and versatile ion-responsive materials-enabled biosensors with high specificity.

## Bio-responsive materials enabled biosensors

Biomolecules serve as the fundamental building blocks that support metabolic processes and the sustenance of life. Thus, diverse technologies and materials have been continually developed to detect biomolecules in a rapid, accurate, and low-cost manner. Bio-responsive materials are among the most appealing types of smart materials due to their biocompatibility, precise controllability, programmability, and efficiency. When exposed to biochemical stimuli like nucleic acids and proteins, bioresponsive materials exhibit a fast response such as phase changes, color changes, fluorescence on/off, or activity switching from inactive to active state. For instance, DNAzyme and CRISPR-Cas can be selectively triggered by biomolecules to exhibit specific catalytic activities, which are thus widely utilized to improve detecting selectivity and accuracy of biosensors [236, 237]. This chapter will center around biomolecule materials that serve as signal transducers or amplifiers in detecting biomarkers in bodily fluids as follows: biomolecule-responsive AIEgens and DNA hydrogels.

### Biomolecule-responsive AIEgens

**Fig. 24:**
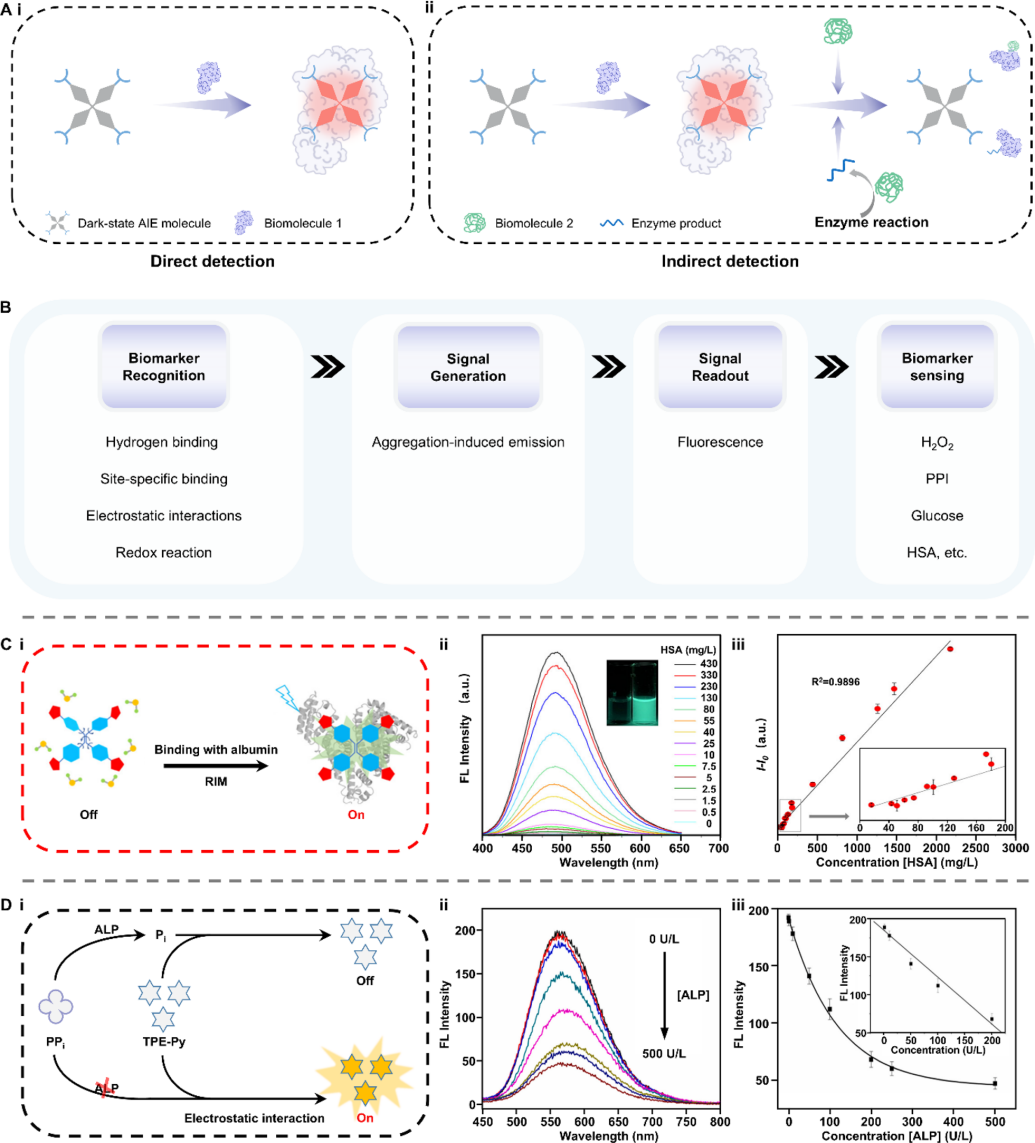
Responsive mechanisms of biomolecule-responsive AIEgens and their working principle when applied in biosensors. **A** Schematic illustrations of **i** direct detection and **ii** indirect detection using biomolecule-responsive AIEgens in biosensing. **B** The working principle underlying biomolecule-responsive AIEgens’ applications in detecting biomarkers in bodily fluids. **C** An AIE-based fluorescent probe for detecting HSA. Schematic illustration of **i** the working principle and **ii** feasibility. **iii** The linear relationship between albumin concentration and the fluorescent intensity (*I*–*I*_*0*_) at 490 nm. Reproduced with permission [248]. Copyright 2019, American Chemical Society. **D** A turn-off strategy to analyze ALP activity. Schematic illustration of **i** the working principle and **ii** feasibility. **iii** The linear relationship between ALP concentration and the fluorescent intensity. Reproduced with permission [249]. Copyright 2023, Elsevier. AIEgens: aggregation-induced emission luminogens; RIM: restriction of intramolecular motion; HSA: human serum albumin; ALP: alkaline phosphatase; PPi: pyrophosphate ion; Pi: phosphate ions; TPE-Py: tetraphenylethene-substituted pyridinium salt

AIEgens serve as powerful analytical tools in biosensing due to their advantages such as low background interference, improved contrast, superior fluorescence lifetime, and good performance in selectivity and long-term monitoring [238]. Similar to ion-responsive AIEgens discussed in Section “Ion-responsive aggregation-induced emission luminogens (AIEgens)”, freely dissolved dark-state AIE molecules can also be aggregated by biomolecules such as proteins to turn on fluorescence. The interaction between biomolecules and free AIE-active molecules dissolved in a solution can be either covalent or noncovalent, including hydrogen bonding, site-specific interactions, and electrostatic binding [239]. Therefore, biomolecule-responsive AIEgens provide a direct way to analyze biomarkers based on the fluorescence-on strategy (Fig. 24A-i). In addition, this fluorogenic aggregation transition can be disrupted or reversed by adding another biomolecule, leading to turning off the fluorescence of light-state AIE molecules (Fig. 24A-ii). Therefore, the FL intensity change of biomolecule-responsive AIEgens can specifically reflect biomolecule concentration in either a direct or indirect way (Fig. 24B) [240–247].

For the direct approach, Tang et al*.* introduced an AIE-based fluorescent probe for detecting human serum albumin (HSA) in complex biological fluids [248]. They found that water-soluble tetrazole-tagged tetraphenylethylene derivatives (TPE-TAs) were specifically bound to albumin through an enthalpy-driven process. This interaction induced a strong turn-on emission, which was then utilized to establish a target-triggered fluorescent biosensor (Fig. 24C-i). The FL intensity of TPE-4TA at 490 nm correspondingly developed with rising HSA concentrations (Fig. 24C-ii). To examine the feasibility of the AIE-based fluorescent assay in clinic diagnosis, they conducted albumin detection using patients’ urine samples, where albumin concentrations were determined by the turbidimetric inhibition immunoassay method and used as references. Results showed that the FL intensity of TPE-4TA/urine mixture (*I*-*I*_*0*_) linearly increased with albumin concentrations, rising from 0.02 to 2500 mg L^−1^ (R^2^ = 0.99) with a low detection limit of 0.21 nM (Fig. 24C-iii).

As a notable example of indirect usage of biomolecule-responsive AIEgens, Liu et al*.* proposed a turn-off strategy to analyze alkaline phosphatase (ALP) activity [249]. Positively charged tetraphenylethene-substituted pyridinium salt (TPE-Py) was used as the AIEgens, which underwent an aggregating process when contacted with the negatively charged pyrophosphate ion (PPi). This electrostatic interaction resulted in the AIE effect, thus enhancing the fluorescence. Therefore, the ALP activity was determined by the TPE-Py/PPi system due to the ALP-enzymatic hydrolysis of PPi into two phosphate ions (Pi), hindering the aggregation of TPE-Py (Fig. 24D-i). As the increase of ALP, the PPi-triggered aggregation weakened, and the AIE effect was diminished correspondingly (Fig. 24D-ii). The ALP concentration in the range of 1–200 U L^−1^ exhibited a linear relationship with the FL intensity of TPE-Py/PPisystem. A LOD of 1 U L^−1^ was achieved (Fig. 24D-iii).

### DNA hydrogels

Nucleic acids play a pivotal role in health monitoring and disease diagnosis, and their abnormal concentrations in bodily fluids are usually connected with disease risk. Consequently, considerable attention and effort have been directed toward nucleic acid-related fields, including synthesis techniques, amplification methods, and detection platforms. The concept of using DNA as building blocks in the development of responsive materials has also gained prominence in response to these needs. The polymeric and hydrophilic nature of DNA enables the binding between DNA and water molecules, leading to the formation of gel-like materials termed DNA hydrogels. These 3D DNA polymer networks inherit both the biological function of DNA and the physical properties of hydrogels. In addition, DNA hydrogels exhibit rapid phase transition in response to certain environmental stimuli such as biomolecules. These charming properties give biomolecule-responsive DNA hydrogels a forefront frontier for biomedical application due to their precise programmability and good biocompatibility [250]. DNA hydrogels are mainly classified into two groups based on the cross-linking and components: pure DNA hydrogel, formed by the crosslinking of DNA itself, and hybrid DNA hydrogels, formed by connecting the DNA molecules that are grafted onto hydrophilic polymer chains [251]. Comprehensive discussions about diverse design principles, synthetic approaches, and functions of biomolecule-responsive DNA hydrogels can refer to previous review articles [252].

**Fig. 25:**
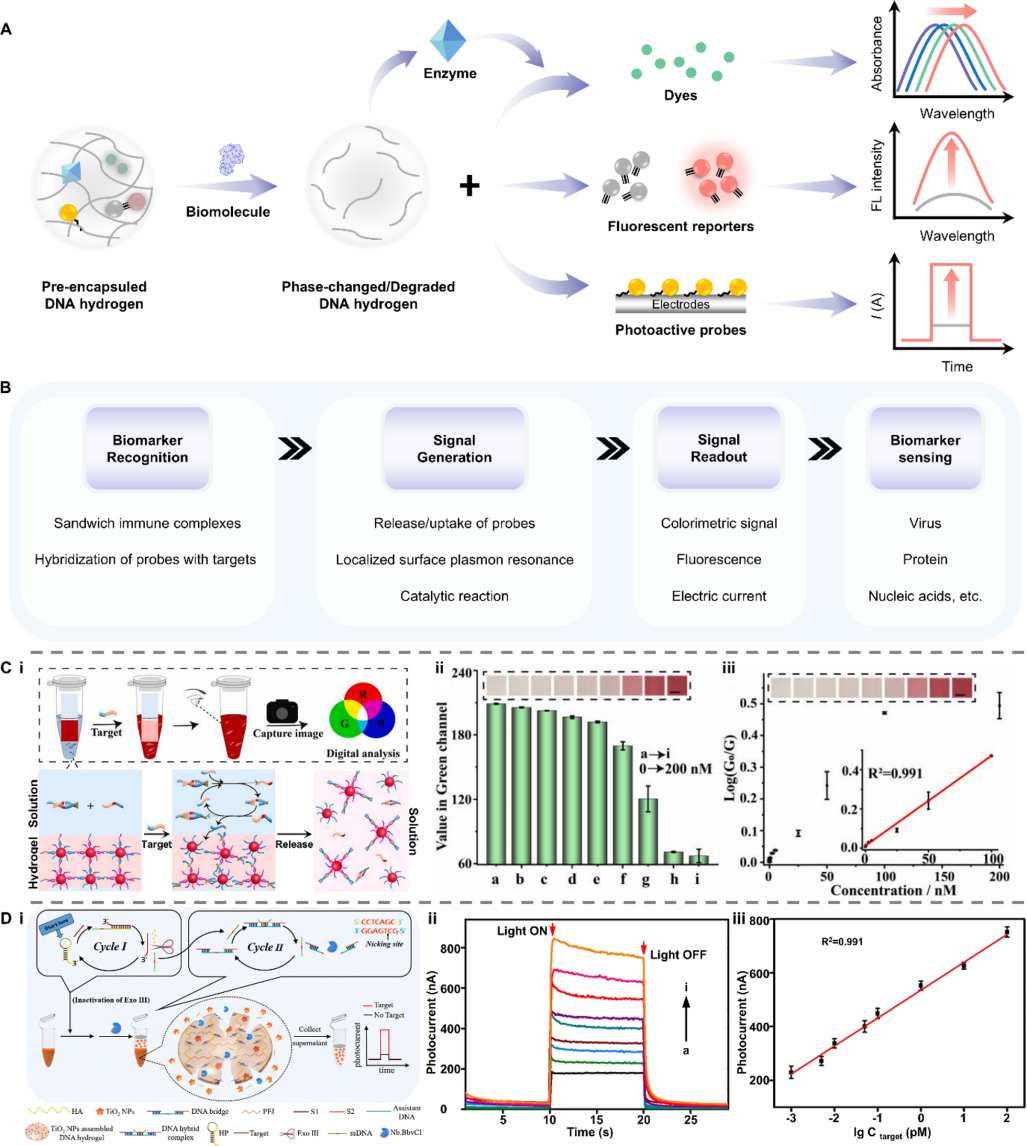
Responsive mechanisms of DNA hydrogels and the working principle when applied in biosensors. **A** Schematic illustrations of biomolecule-responsive mechanisms for constructing colorimetric, fluorescent and PEC biosensors. **B** The working principle underlying DNA hydrogels’ applications in detecting biomarkers in bodily fluids. **C** A colorimetric biosensor for microRNA detection based on DNA-AuNP hybrid hydrogel. **i** Schematic illustration of the working principle. **ii** The value in the green channel of solutions with different miRNA-21 concentrations. **a**–**i** 0 nM; 0.05 nM; 0.5 nM; 2.5 nM; 5 nM; 25 nM;50 nM; 100 nM; 200 nM). **iii** The linear relationship between miRNA-21 concentration and the logarithm of the relative green value (Log(G_0_/G)). Reproduced with permission [260]. Copyright 2023, Elsevier. **D** A PEC biosensor for miRNA analysis using TiO_2_NP-embedded DNA hydrogels. **i** Schematic illustration of the working principle. ii) The PEC signal of the target at various concentrations **a**–**i** 0 fM; 1 fM; 5.0 fM; 10 fM; 50 fM; 100 fM; 1.0 pM; 10 pM; 100 pM). **iii** The linear relationship between the logarithmic concentration of the miRNA-155 and the photocurrent value. Reproduced with permission [261]. Copyright 2021, Springer Vienna. AuNP: gold nanoparticle; PEC: photoelectrochemical

In the presence of specific biomolecules, biomolecule-responsive DNA hydrogels exhibit a sol–gel conversion and release pre-encapsulated report probes to detect target biomarkers (Fig. 25A). For example, fluorescent indicators such as QDs are released when DNA hydrogels dissolve by target DNA, leading to a fluorescent intensity change of the whole solution system for visual analysis [253]. Also, coated fluorescent probes can be activated by escaping from DNA hydrogels to detect target DNA based on the FL intensity change. Moreover, target DNA-triggered collapse of DNA hydrogel facilitates the contact between photoactive probes and electrodes, increasing electric current to quantify biomarkers [254]. This specific DNA-induced phase transition allows DNA hydrogel to serve as desirable signal transducers to construct colorimetric, fluorescent, and PEC biosensors (Fig. 25B) [255–259].

Using the phase transition principle, Guo et al*.* established a portable colorimetric testing platform for miRNA detection by employing the DNA-AuNP hybrid hydrogel [260]. When exposed to specific miRNA, the trigger DNA was activated and amplified to disintegrate the hybrid hydrogel film. As a result, AuNPs were released from the hydrogel film into the solution, leading to a color change for digital image colorimetric analysis (Fig. 25C-i). The value in the green channel (G) of the solution correspondingly diminished as the miRNA-21 concentration rose from 0 to 200 nM (Fig. 25C-ii). A good linearity was found between the miRNA-21 concentration ranging from 0.05 to 100 nM and the logarithm of the relative G value (Log(G_0_/G)) (R^2^ = 0.991), with a LOD of 17.0 pM (Fig. 25C-iii). In another approach, Liu et al*.* constructed a PEC biosensor for miRNA analysis using TiO_2_ NP-embedded DNA hydrogels [261]. In the presence of target miRNA, TiO_2_ NPs were released to the supernatant, which was further collected for the PEC test (Fig. 25D-i). The value of the photocurrent increased synchronously with the rise of miRNA-155 concentration (Fig. 25D-ii). A good linear relationship was obtained between photocurrent value (*I*) and the logarithmic concentration of the miRNA-155 (C_target_) from 1.0 fM to 100 pM (R^2^ = 0.991), with a LOD of 0.41 fM (Fig. 25D-iii).

These developments demonstrate the growing capabilities that bioresponsive materials can be incorporated into biosensors for preparing portable and accurate biosensors and for making what conventionally impossible to be possible to detect some biomarkers. Of particular interest would be the development of stable bioresponsive materials that can be maintained at different environmental temperatures for a long term and efficient bioresponsive materials that can respond and report target analytes at a low concentration without additional amplification process.

## Conclusions and perspective

Stimuli-responsive smart materials have demonstrated immense potential for incorporation into biosensors for liquid biopsies, particularly in the detection and quantification of biomolecules. These materials have shown the ability to enhance the sensitivity, accuracy, and functionality of biosensors, as summarized below:

- The barrier-breaking effect of ultrasound-responsive micro- and nanobubbles facilitates the release of extratemporal biomarkers into bodily fluids, such as plasma. This barrier-breaking effect significantly enhances the accuracy and sensitivity of liquid biopsies, enabling more reliable detection of disease biomarkers.
- MOFs encapsulated with multiple QDs are instrumental in constructing ratiometric biosensors. These biosensors offer built-in self-calibration mechanisms for signal correction, improving precision and user-friendliness by minimizing external interferences and variability in results.
- Magnetic and electro-responsive materials support the fabrication of compact and portable biosensors due to their low cost, wide availability, and ease of integration with existing technologies. These materials are particularly valuable for point-of-care applications.
- Materials responsive to specific ions or biomolecules provide a direct, biocompatible approach for biomarker detection in bodily fluids. Their high specificity ensures accurate identification and quantification of targets in complex biological environments.
- Thermo-responsive materials enable detection based on temperature variations and have potential applications in monitoring heat-sensitive biomarkers.

Despite the remarkable potential of stimuli-responsive smart materials in advancing biosensor technology, addressing the following challenges is essential to unlock their full potential:

- Precise and localized manipulation of ultrasound-responsive microbubbles and microrobots at the micro- and nanoscale currently depends on high-power and complex ultrasound systems. Additionally, the interaction mechanisms between acoustic waves and micro/nanomaterials remain insufficiently understood, limiting their optimization and application.
- Light- and electro-responsive materials are prone to non-specific binding, which reduces their specificity and functionality when detecting multiple biomarkers in complex liquid samples. Strategies to mitigate such interference are essential for their broader adoption.
- Magnetic materials are highly sensitive to factors such as size, shape, and external magnetic fields. Achieving high uniformity in magnetic properties, size, and shape remains challenging and requires careful design and synthesis strategies.
- Current thermo-responsive materials lack the precision to provide real-time feedback on minor temperature changes, which is crucial for detecting subtle thermal variations associated with biomarker interactions.
- Smart materials derived from biological molecules often exhibit limited thermal stability and efficiency, posing challenges for mass production and widespread adoption. Enhancing their stability and scalability is critical for their practical applications.
- To advance point-of-care and at-home diagnostics, further attention should be given to miniaturizing sample processing, signal transduction, and detection systems (e.g., ultrasound generation/control, optical detection, and magnetic detection systems). These components must be seamlessly integrated into portable devices at low cost to ensure ease of use and accessibility in real-world settings.
- The combination of dual or multiple stimuli-responsive systems (e.g., ultrasound-light or magnetic-thermal) may offer significant potential to synergistically enhance the detection process. Such hybrid systems could enable faster, more accurate, and multifunctional diagnostic capabilities, particularly when analyzing patient samples with complex compositions. For instance, integrating ultrasound systems with magnetic-responsive subsystems could simplify biomarker detection through wash-free approaches, reducing the complexity and time required for analysis while improving overall diagnostic efficiency.

For more examples, we encourage the reader to refer to Table 1 that provides a comprehensive comparison between existing biosensors for liquid biopsies using various stimuli-responsive smart materials in terms of their sensing mechanisms, clinical application, and performance, including sensitivity, LOD, linear range (LR) and fluorescence enhancement factors.

**Table 1:** Stimuli-responsive smart materials enabled biosensors for liquid biopsies

Type	Materials	Target analyte	Mechanism	LOD and LR	Signal/detecting enhancement factor	Refs.
Mechano-responsive materials Enabled Biosensors	Micro-and nanobubbles	miRNA	US-induced BBB opening	N/A	10.9-fold compared to control groups (TA)	[22]
		miRNA	US-induced BBB opening	N/A	221-fold compared to control groups (TA)	[35]
		ctDNA	US-induced BBB opening	N/A	270-fold in pigs compared to static conditions (TA)	[43]
	Acoustic nanorobots	miRNA	US propulsion	N/A	17-fold compared to static conditions (FI)	[55]
		miRNA	US propulsion	N/A	2.3-fold compared to static conditions	[61]
		HAS	US streaming	0.5 μg mL^−1^ 0–30 μg mL^−1^	200-fold compared to conditional detecting methods	[58]
		Rabbit IgG	US aggregation	0.58 ng mL^−1^ 1–20 ng mL^−1^	34.5-fold compared to conventional LFIA	[59]
		Tau protein	US aggregation	10.30 pg mL^−1^ 0–0.4 ng mL^−1^	Threefold compared to conventional ELISA	[60]
		CEA	US aggregation	0.012 ng mL^−1^ 0.1–20 ng mL^−1^	Tenfold compared to control groups	[62]
	Piezoelectric materials	Blood pressure	Piezoelectric effect	Sensitivity: 0.062 kPa^−1^	Threefold compared to their previous studies	[65]
		Blood pressure	Piezoelectric effect	Sensitivity: 0.095 mV mmHg^−1^	N/A	[66]
		Blood pressure	Piezoelectric effect	Accuracy: ^a^Grade A level	Accuracy compared to the professional medical equipment	[67]
	Liquid metals	Blood pressure	Ohm's law	Sensitivity: 0.158 kPa^−1^ LR: 16–600 kPa	Accuracy compared to the professional medical equipment	[79]
Light-Responsive Materials Enabled Biosensors	Artificial enzyme mimics	Urease	Photocatalysis	3.1 uM 10–500 uM	Threefold compared to other fluorescent assays	[86]
	Artificial enzyme mimics	GSH	Photocatalysis	0.06 μM 0.2–20 μM	Tenfold compared to previous colorimetric assays	[87]
		GSH	Photocatalysis	0.68 μM 0–20 μM	N/A	[93]
		ACP	Photocatalysis	0.0415 U L^−1^ 0.05–2.5 U L^−1^	Shorter detecting time	[99]
		GSH	Photocatalysis	0.225 μM 0.4–60 μM	Wider detecting range	[101]
		miRNA	Photocatalysis	44.76 fM 50–3000 fM	N/A	[102]
	Quantum dots	ATP	Exciton energy transfer	0.1 pM 0.2 pM–1 μM	100-fold compared to commercial ELISA assays	[129]
		ssDNA	FRET	50 pM 0.5–5 nM	N/A	[130]
		METTL3/14 complex	FRET	0.0311 fM	10^5^-fold compared to previous ELISA assays	[131]
	Metal–organic frameworks	IL-6	Quenching of FRET	70 pM 0–14.4 nmol L^−1^	N/A	[136]
		Arginine	FRET	4.87 μM	Fivefold compared to other fluorescent assays	[139]
		GSH	FRET	0.90 nM 3–25 nM	50- to 650-fold compared to other fluorescent assays	[140]
		AFP	Steric hindrance	0.16 pg mL^−1^ 0.002–15.0 ng mL^−1^	3- to 200-fold compared to conventional assays	[141]
	Plasmonic nanoparticles	AIMP-2	MEF	0.007 ng mL^−1^ 0.01–10000 ng mL^−1^	2.5-fold compared to planar Au (FI)	[148]
		PSA	SERE	0.1 ng mL^−1^, 0.1–20 ng (f-PSA) 0.7 ng mL^−1^, 1–200 ng (t-PSA)	Accuracy compared to other immunosensing assays	[149]
		miRNA	LSPR	LOD at low nM level	N/A	[150]
Electro-responsive materials enabled biosensors	Piezoelectric materials	virus, nucleic acid, ect	Eclectic signal change by mass change	LOD at pg mL^−1^ level	Accuracy and sensitivity compared to those of gold standard techniques such as ELISA and PCR; selectivity compared to surface plasmon resonance and potentiometric biosensors	[159–161]
	Conductive polymers	virus, DNA, GSH, ion, ect	Electrochemical	LOD at pM level	Accuracy and sensitivity compared to those of gold standard techniques such as ELISA and PCR,	[166, 174, 175]
Magnetic-responsive materials enabled biosensors	Magnetic nanoparticles	virus, DNA, PSA, ect	Magnetoresistive	LOD at nM level	Sensitivity compared to ELISA	[184–186]
		virus	Brownian relaxation	0.084 nM (5.9 fmol)	Sensitivity compared to ELISA	[194]
		Streptavidin	Brownian relaxation	75 nM (7.5 pmol)	N/A	[195]
		H1N1 nucleoprotein	Brownian relaxation	44 nM (4.4 pmol)	Sensitivity compared to the fluorescent assays	[196]
		CEA, PSA, etc	Magnetoresistive	0.27 ng mL^−1^ (CEA), 0.02 ng mL^−1^ (t-PSA), 0.07 ng mL^−1^ (f-PSA)	N/A	[198]
		SARS-CoV-2 spike and nucleocapsid proteins	Brownian relaxation	1.56 nM (125 fmole) and 12.5 nM (1 pmol) for SARS-CoV-2 spike and nucleocapsid proteins	Sensitivity compared to qPCR and fivefold compared to antigen detection	[199]
		SARS-CoV S antibody	Brownian relaxation	2 ng mL^−1^ (0.33 fmol)	100-fold and tenfold compared to flow cytometry devices and ELISA tests, respectively	[200]
Thermo-responsive materials enabled biosensors	Thermochromic materials	cTnI protein	Thermochromics	0.021 ng mL^−1^ 0.05–20 ng mL^−1^	Compared to other portable sensors	[211]
Ion-responsive materials enabled biosensors	pH-responsive chromophores and fluorophores	Ach	Fluorescence quenching	LOD:16.28 µM and 0.29 nM, LR: 20–200 µM and 1–100 nM, respectively	N/A	[221]
		urease	Inner filter effect	LOD: 1.67 L^−1^ (fluorometric) and 1.07 U L^−1^ (colorimetric), LR: 2–40 U L^−1^	N/A	[222]
		urea	Formation of fluorophores	0.0103 mM 0.02–20 mM	N/A	[223]
	Ion-responsive AIEgens	AFP	Ag^+^-driven AIE	42 pg m L^−1^ 0.1–5000 ng m L^−1^	4- to 100-fold compared to other fluorescence immunoassays	[232]
		ALP	Al^3+^-driven AIE	0.15 U L^−1^ 0.5–25 mU m L^−1^	2- to tenfold compared to other fluorescent assays	[234]
		Glucose	Ce^3+^-driven AIE	2.4 μM 8–48 μM	3- to 100-fold compared to other fluorescent assays	[235]
Bio-responsive materials enabled biosensors	Biomolecule-responsive AIEgens	Nucleic acid	Crispr-driven AIE	1 fM 1–105 fM	80-fold more sensitive compared to the traditional FQ reporter-based Crispr-Dx without amplification	[241]
		HAS	P_3_-COOH-driven AIE	56 nM 0.1–2.5 μM	131-fold compared to other hyperbranched probes (FI)	[247]
		Albumin	Albumin-driven AIE	0.21 nM 0.02–3000 mg L^−1^	The best LOD value in the reported fluorogenic albumin probes so far	[248]
	DNA hydrogels	cfDNA	Catalytic reaction	0.042 pM 0.1 pM-1500 nM	Tenfold to 200-fold compared to other colorimetry and fluorescent assays	[256]
		miRNA	Release of AuNPs	86.0 fM 0.25 pM-2.5 nM	N/A	[260]

LOD: limit of detection; LR: linear range; miRNA: microRNA; US: ultrasound; BBB: blood–brain barrier; TA: the level of target analyte; N/A: not available; ctDNA: circulating tumor DNA; FI: the level of fluorescence intensity; HAS: human serum albumin; LFIA: lateral flow immunoassay; ELISA: enzyme linked immunosorbent assay; CEA: carcinoembryonic antigen; GSH: glutathione; ACP: acid phosphatasel; ATP: adenosine 5'-triphosphate; ssDNA: single strand DNA; FRET: fluorescence resonance energy transfer; METTL3/14 complex: methyltransferase-like 3 (METTL3) and methyltransferase-like 14; IL-6: Interleukin-6; AFP: alpha-fetoprotein; AIMP-2: aminoacyl-tRNA synthetase complex interacting multi-functional protein 2; MEF: metal-enhanced fluorescence; PSA: prostate-specific antigen; f-PSA: free prostate-specific antigen; SERE: Surface enhanced raman scattering; LSPR: localized surface plasmon resonance; t-PSA: total prostate-specific antigen; PCR: polymerase chain reaction; qPCR: real-time quantitative polymerase chain reaction; Ach: acetylcholine; cTnI protein: cardiac troponin I protein; AIE: aggregation-induced emission; FQ reporter: fluorescently quenched reporter; cfDNA: cell-free DNA

^a^Grade A level: according to the British Hypertension Society (BHS) standard

From a clinical perspective, transitioning smart material-enabled biosensors from research laboratories to practical clinical and home applications depends on optimizing their detection performance and translational potential. To enhance clinical relevance and usability, biosensors must reliably detect target analytes at clinically significant concentrations while minimizing false positives and negatives. Consistent performance over extended periods is essential, particularly for continuous or repeated testing in both clinical and home settings. In addition, user-friendly protocols that minimize sample preparation are critical in point-of-care and home-testing scenarios, where complex procedures can hinder adoption and introduce user error.

Another critical consideration for clinical use is the protein corona effect [262]. When nanoparticles or materials interact with biological fluids (e.g., plasma or saliva), proteins and other biomolecules quickly adsorb onto their surfaces, forming a corona. This biological layer modifies the material's physicochemical properties, significantly impacting its performance in biosensing applications. While the protein corona can impede biosensor function by masking recognition sites, lowering sensitivity, and causing non-specific interactions that lead to false positives or variability, it also presents potential opportunities. Differences in corona composition could serve as indicators of disease-specific biomarker patterns, offering new avenues for diagnostic development [262].

In summary, the future of smart material-enabled biosensors in academia possesses immense potential to revolutionize diagnostics and transition into commercially viable devices. However, translating these innovations into practical applications requires interdisciplinary collaboration in material science, engineering, and healthcare. Efforts must also address scalability, regulatory approval processes, and compatibility with existing diagnostic workflows.

## Data Availability

No datasets were generated or analysed during the current study.
